# A genetic screen to discover SUMOylated proteins in living mammalian cells

**DOI:** 10.1038/s41598-017-17450-7

**Published:** 2017-12-12

**Authors:** Maki Komiya, Akihiro Ito, Mizuki Endo, Daisuke Hiruma, Mitsuru Hattori, Hisato Saitoh, Minoru Yoshida, Takeaki Ozawa

**Affiliations:** 1 Department of Chemistry, Graduate School of Science, The University of Tokyo, 7-3-1 Hongo, Bunkyo-ku, Tokyo, 113-0033 Japan; 2 Chemical Genetics Laboratory, RIKEN, 2-1 Hirosawa, Wako, Saitama, 351-0198 Japan; 3 Chemical Genomics Research Group, RIKEN Center for Sustainable Resource Science, 2-1 Hirosawa, Wako, Saitama, 351-0198 Japan; 4 Department of Biological Sciences, Graduate School of Science and Technology, Kumamoto University, 2-39-1 Kurokami, Kumamoto, 860-8555 Japan; 5 Present Address: Department of Biomolecular Science and Engineering, The Institute of Scientific & Industrial Research, Osaka University, Osaka, Japan

## Abstract

Post-translational modification by the Small Ubiquitin-related Modifier (SUMO) is indispensable for diverse biological mechanisms. Although various attempts have been made to discover novel SUMO substrate proteins to unveil the roles of SUMOylation, the reversibility of SUMOylation, and the differences in the SUMOylation level still makes it difficult to explore infrequently-SUMOylated proteins in mammalian cells. Here, we developed a method to screen for mammalian SUMOylated proteins using the reconstitution of split fluorescent protein fragments in living mammalian cells. Briefly, the cells harboring cDNAs of SUMOylated proteins were identified by the reconstituted fluorescence emission and separated by cell sorting. The method successfully identified 36 unreported SUMO2-substrate candidates with distinct intracellular localizations and functions. Of the candidates, we found Atac2, a histone acetyltransferase, was SUMOylated at a lysine 408, and further modified by multiple SUMOs without isoform specificity. Because the present method is applicable to other SUMO isoforms and mammalian cell-types, it could contribute to a deeper understanding of the role of SUMOylation in various biological contexts.

## Introduction

Once certain proteins are translated from mRNAs, they are further modified by small molecules via covalent conjugation to modulate their functions. Of the post-translational modifiers, proteins called Small Ubiquitin-related Modifiers (SUMOs) diversely regulate many cellular biological events using unique reaction modes^1,2^. Mammalian cells express at least three different SUMO isoforms^1–5^. SUMOs are covalently attached to lysine residues in the substrate proteins by sequential enzymatic reactions with E1 (an ATP-dependent SUMO-activating enzyme), E2 (a SUMO-conjugating enzyme), and E3 (a SUMO ligase)^1,2,6^. Each SUMO isoform has a different substrate selectivity^7,8^ and conjugation mode: RanGAP1, the first reported SUMO protein substrate^9^, was reported to be preferentially SUMOylated by SUMO1, which contributed to the protein’s stability^10^, and amyloid β peptide generation was reduced by polySUMO chain formation by SUMO3^11^. The fraction of SUMOylated proteins is normally less than 1% under normal conditions^1,12^ and is strictly regulated by a balance between SUMOylation and deSUMOylation that is mediated by a SUMO-specific isopeptidase^13^. Although the SUMOylated fraction is small, modification by SUMO is indispensable for various biological mechanisms, including DNA repair, cell cycle, and signal transduction^1,2,12,14–19^.

Various attempts have been made to discover novel SUMOylated proteins to unveil the roles of SUMOylation in biological events. However, the detection of SUMOylated proteins is sometimes difficult because target proteins are rarely SUMOylated and are rapidly deSUMOylated upon cell lysis by SUMO-specific proteases^1^. For example, in a previous screening method that was based on immunoprecipitation, SUMOylated proteins were collected from cell lysates and then analyzed using mass spectrometry (IP-MS)^20,21^. However, because of the difficulty in completely inhibiting de-SUMOylation during immunoprecipitation, the IP-MS method preferentially detected proteins that might be frequently SUMOylated and resistant to deSUMOylation. Therefore, the scope of the SUMOylation candidates was biased. A system based on yeast two-hybrid screening was developed to detect SUMOylated proteins in living yeast to overcome the difficulties with cell lysis^22^. This two-hybrid screen is useful, but this method still has some difficulties in detecting mammalian SUMOylated proteins. First, the yeast SUMOylation system might be too simple to satisfactorily explore mammalian SUMOylation because yeast cells express only one SUMO isoform^1,12,23,24^; in contrast, mammalian cells have at least three SUMO isoforms, each with different substrate selectivity. Second, mammalian SUMOylation patterns that differ by cell type cannot be examined using the yeast system^25,26^. Third, yeast two-hybrid screening requires that the candidate proteins are translocated to the nucleus, which biases the selection of the substrate proteins. Because of these issues, a non-destructive screening method is required to identify novel mammalian SUMO substrate proteins in living mammalian cells.

We herein present a novel system for the screening of SUMOylated proteins. To detect SUMOylation in living mammalian cells, we reconstituted split fluorescent protein fragments^27–30^. Because the reconstitution of split fluorescent protein fragments is irreversible and occurs without destroying the cell, it is suitable for the detection of less abundant SUMOylated proteins. By combining this method with the use of fluorescence-activated cell sorting (FACS), which automatically collects fluorescent cells, we can collect cells that contain SUMOylated proteins in a high-throughput manner. Using this system, we have succeeded in identifying new mammalian SUMOylated protein candidates, especially those targeted by SUMO2, and have discovered that Atac2 was SUMOylated by SUMO2 at a lysine 408, both *in vivo* and *in vitro*.

## Results

### Detection of SUMOylation by SUMO2 using reconstitution of the split Venus fragments

**Figure 1:**
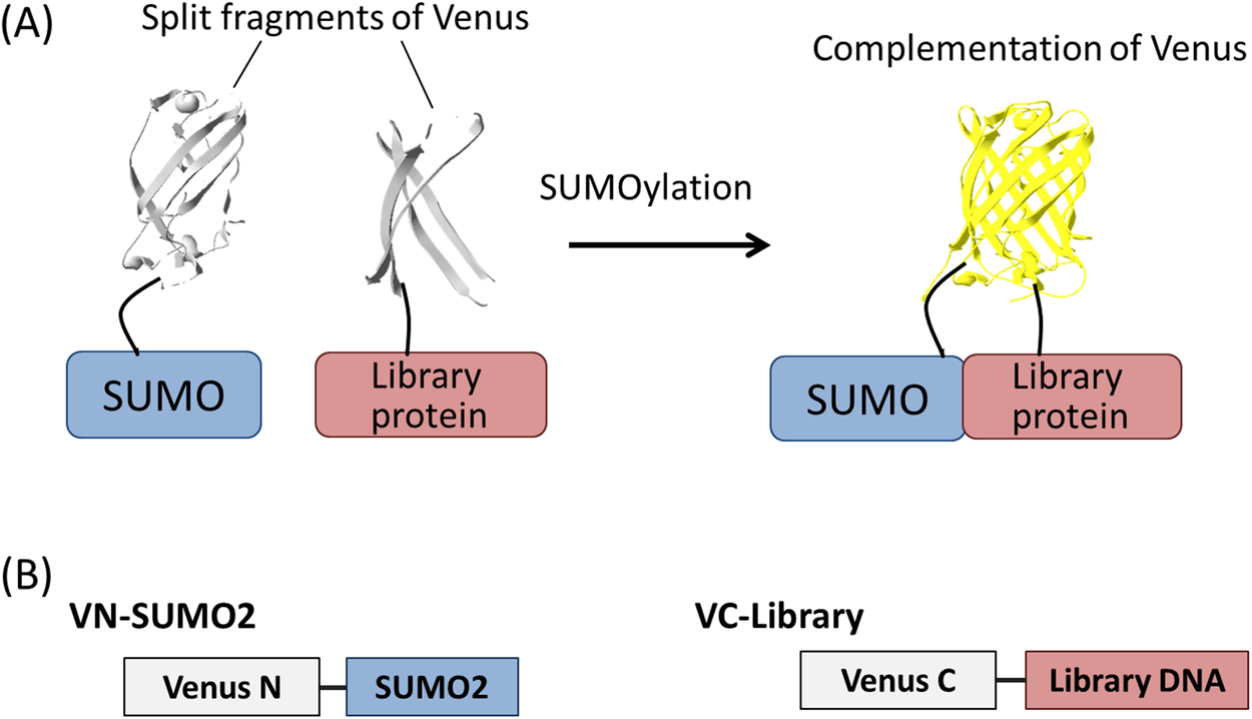
The probes for detecting SUMOylation in living cells. (**A**) Schematic for detecting SUMOylation under live-cell conditions using the reconstitution of split fluorescent protein fragments. (**B**) Schematic structures of VN-SUMO2 and VC-library probes. Venus N: N-terminal fragment (amino acids 1 to 158) of Venus. Venus C: C-terminal fragment (amino acids 159 to 240) of Venus (VC).

Unlike SUMO1, SUMO2/3 form polySUMO chain and have reactivity to extracellular stimuli^31^. Because of these unique SUMOylation features and its potential for future analysis, we selected SUMO2 as a first demonstration for the screening of SUMOylated proteins. The N-terminal fragment of Venus, a yellow fluorescent protein with bright fluorescence^32^, was fused with the N-terminus of SUMO2 by a flexible GS linker (Gly-Gly-Gly-Gly-Ser) to make VN-SUMO2 (Fig. 1). The C-terminal Venus fragment was fused to the proteins that were encoded by the randomly extracted mouse cDNA library to make VC-library. If the VC-library proteins are modified by VN-SUMO2 in living cells, the two Venus fragments are brought close together, which results in the recovery of fluorescence. The fluorescent cells were screened and collected from a population of the cells using a cell sorter.

**Figure 2:**
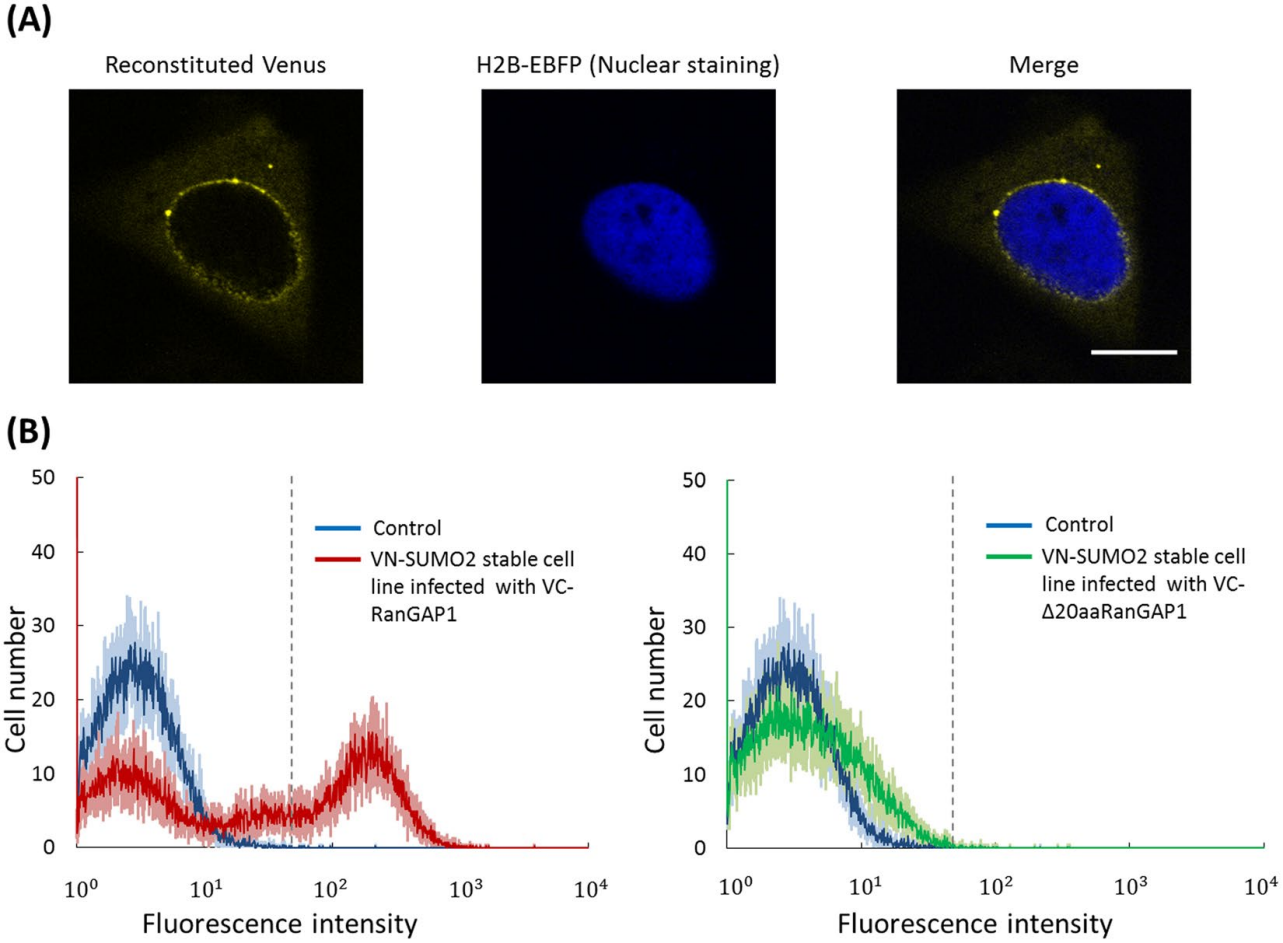
Evaluation of the probes by using SUMOylated protein RanGAP1. (**A**) Fluorescence images of the VN-SUMO2 stable cell lines transiently expressing VC-RanGAP1 and H2B-EBFP. Scale bar: 10 μm. (**B**) Fluorescence intensities of the non-infected VN-SUMO2 stable cell lines (control cells) and the VN-SUMO2 stable cell lines infected with VC-RanGAP1 (left) or VC-Δ20aaRanGAP1 (right) were analyzed by FACS. Each histogram was obtained from measurements of 5,000 cells and the measurements were repeated 5 times. Blue, red, and green lines indicate the average of the 5 measurements of control cells, VC-RanGAP1-infected cells, and VC-Δ20aaRanGAP1-infected cells, respectively. Light blue, light red, light green indicate the respective standard deviation. Dotted gray lines indicate the point where cell number of control cells is almost zero.

First, murine NIH3T3 cell lines that stably express VN-SUMO2 were generated. The well-known SUMO substrate protein RanGAP1^9,33–35^ was used to confirm that the Venus fluorescence is recovered when a specific target is SUMOylated. Human RanGAP1 was fused with the C-terminal Venus fragment (VC-RanGAP1). A deletion mutant of RanGAP1 that lacks 20 amino acids flanking the K524 SUMOylation site^34^ was also fused to the Venus fragment (VC-Δ20aaRanGAP1) and was used as a negative control. The amino acid sequences that flank the RanGAP1 SUMOylation site are recognized by SUMO modification machinery^36^. A high intensity of fluorescence from Venus was detected at the periphery of the nucleus in cells that co-expressed VC-RanGAP1 and VN-SUMO2 (Fig. 2A). The RanGAP1 localization was consistent with that of a previous report that indicated that SUMOylated RanGAP1 translocated from the cytosol to the nuclear membrane^35^. Therefore, the localization of the reconstituted Venus suggested that SUMOylation of RanGAP1 was specifically visualized in living cells.

Next, cDNA of VC-RanGAP1 or VC-Δ20aaRanGAP1 was introduced via retrovirus into the VN-SUMO2 stable cell lines, and the fluorescence intensities of the infected cells were analyzed using a FACS (Fig. 2B). We calculated the percentage of the infected cells which showed higher fluorescence intensities than the maximum intensity of the control cells (Fig. 2B, a dotted gray line) to the whole cells. A 43(±3)% population of the VC-RanGAP1-infected cells displayed higher fluorescence intensities (Fig. 2B, Left), indicating that the reconstituted Venus protein produced sufficient fluorescence intensity for FACS analysis. The population of VC-Δ20aaRanGAP1-infected cells showed almost non-fluorescence but some population distributed relatively to higher fluorescence intensity area compared to that of the control cells, due to the presence of false positives. Only 0.16(±0.03)% of the VC-Δ20aaRanGAP1-infected cells surpassed the maximum fluorescence intensity of the control cells (Fig. 2B, Right). This indicates that the maximum fluorescence intensity of the control cells was appropriate threshold to discriminate SUMOylated proteins from non-SUMOylated proteins for the subsequent sorting. We thus concluded that the SUMOylation of target proteins can be determined using fluorescence microscopy and FACS analysis to detect the reconstitution of the Venus fragments.

### Identification of the SUMOylated protein candidates

**Figure 3:**
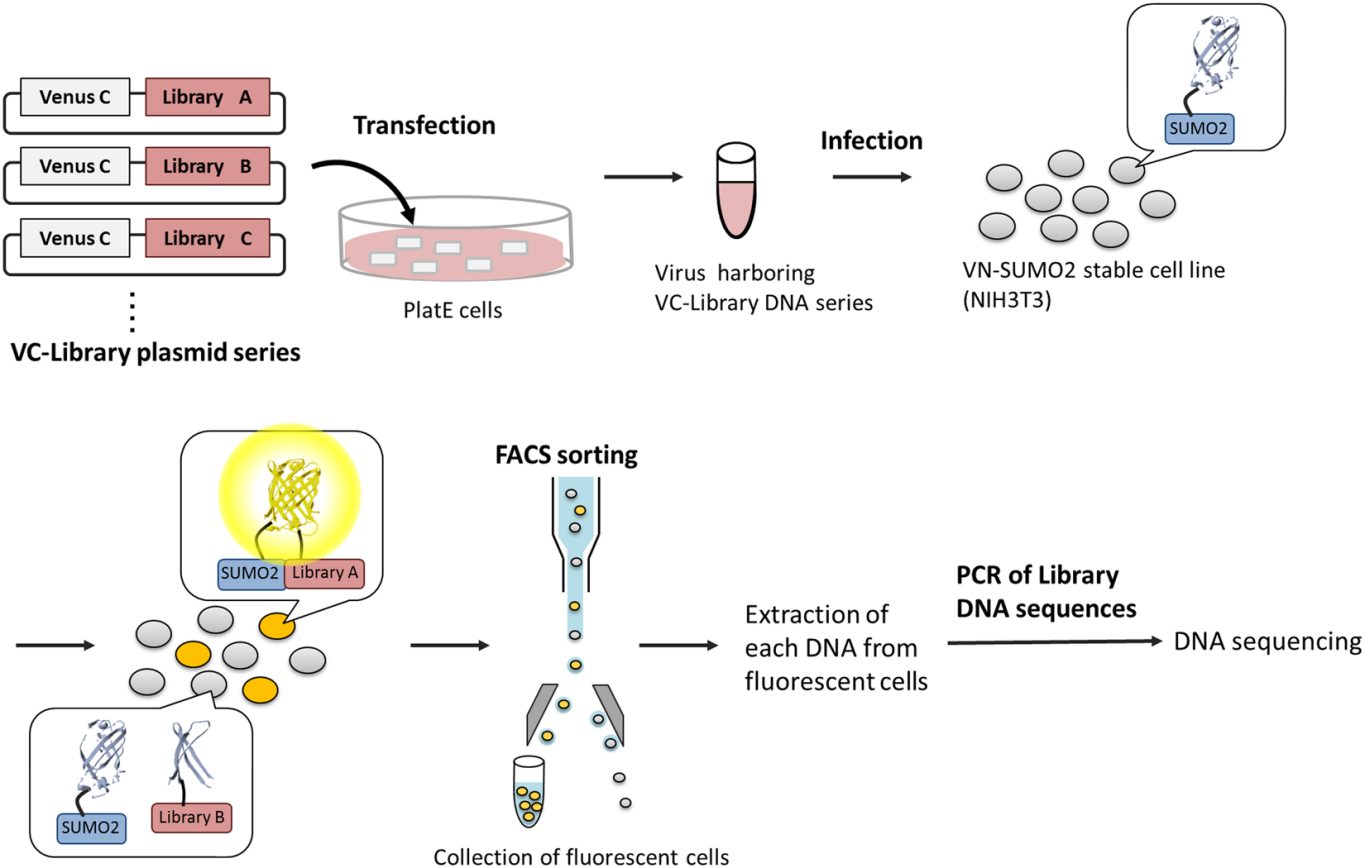
Schematic of screening mammalian SUMOylated proteins based on the reconstitution of split Venus fragments. Library DNAs are inserted into virus infection vectors with DNA of VC fragment and transfected into PlatE cells. The produced viruses harboring VC-library DNAs are added to NIH3T3 cells that stably express VN-SUMO2. The fluorescent cells harboring reconstituted Venus are sorted by FACS. The library DNA is extracted from each fluorescent cell. SUMOylated protein candidates are identified by an analysis of the extracted DNA sequences.

**Figure 4:**
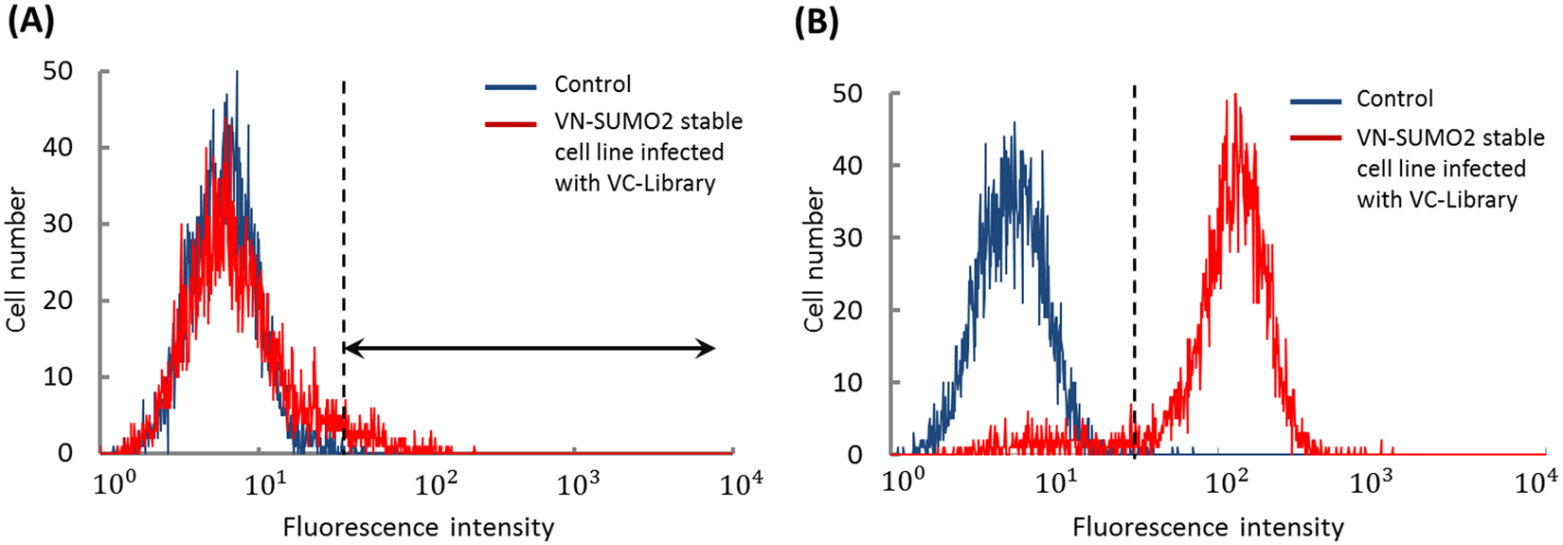
FACS isolation of the fluorescent cells. Fluorescence intensities of the non-infected VN-SUMO2 stable cell lines (control cells) and the VN-SUMO2 stable cell lines that were infected with VC-library DNAs (probe-introduced cells) were analyzed by FACS. (**A**) Comparison of control cells with the probe-introduced cells before FACS sorting. The region indicated with an arrow includes the cells that have higher fluorescence intensities than control cells. (**B**) Comparison of control cells with the probe-induced cells after FACS sorting. The target fluorescent cells were repeatedly incubated and sorted by a FACS four times. The data show the fluorescence intensity of the cells after the last sorting.

Next, we applied this method to screen mammalian SUMOylated protein candidates (Fig. 3). The NIH3T3 cells that stably expressed VN-SUMO2 were infected with the retrovirus that harbored cDNA libraries that were fused with the cDNA of VC proteins (VC-library). The infection efficiency was increased to up to 30% to introduce a single piece of VC-library DNA into each NIH3T3 cell. The percentage was estimated using FACS analysis with GFP-infected NIH3T3 cells under the same infection conditions (Supplementary Fig. 1). After VC-library-infected cells were incubated for a few days, some of the cells fluoresced more intensely than did the control cells (Fig. 4A). This result suggested that some of the VC-library proteins were modified by VN-SUMO2, which subsequently reconstituted the Venus protein, causing fluorescence emission. The fluorescent cells were sorted by FACS and incubated for a week to increase their population. Subsequently, the fluorescent cells were sorted again to increase the accuracy of the fluorescent cell collection. The sorting and incubation process was repeated 3–4 times. FACS analysis of the cell population that was finally obtained resulted in the accurate separation of the fluorescent cells (Fig. 4B). The individual fluorescent cells were then separately plated on a culture dish, and the single-cell clones were isolated. From the DNA sequence analysis of the cDNAs that were extracted from the isolated cells, we identified 38 SUMOylated protein candidates (Table 1). Among the identified candidates, 17 proteins harbored SUMO consensus recognition sequences, Ψ-K-X-E/D (“Ψ”: a hydrophobic amino acid, “K”: the SUMO-modified lysine residue, “X”: one of any amino acids, “E”: a glutamic acid, “D”: an aspartic acid), which indicated their potential SUMOylation sites (Table 2 and Supplementary Table 2). Based on previous reports, the screened candidate proteins are localized in various intracellular compartments: Anxa5 in the cytoplasm and the nucleus^37^, Drosha in the nucleus^38^, and Plscr3 and Tuba1b in mitochondria and microtubules, respectively^39,40^. The functions of the identified candidate proteins are also diverse: Narf is an ubiquitin ligase^41^, Myof regulates membrane integrity to affect vascular endothelial growth factor signaling^42^, and Arpc1b and Taz are related to cell cycle progression and cell proliferation, respectively^43,44^. This diversity indicated that the present method can screen various SUMOylated protein candidates without bias in terms of their normal intracellular localization and function in living mammalian cells. Of the candidates, two proteins, Rpl37a and Lmna, have already been reported to be modified by SUMO2 in mammalian cells^45,46^, which confirms that the present method can identify SUMOylated proteins in living cells. Given that the method could also detect SUMO-interacting proteins, which was implied by the presence of putative SUMO-interacting motifs (SIM), such as [V/I]-X-[V/I]-[V/I] (“V”: valine, “I”: isoleucine, “X”: one of any amino acids)^47^, in some candidates (Table 2 and Supplementary Table 2), further analysis is required to confirm their SUMOylation. Consequently, the screening method has identified 36 proteins that have not been reported to be SUMOylated as SUMOylation candidates.

**Table 1:** The SUMOylated protein candidates identified by cDNA analysis of the fluorescent cells sorted by FACS.

**Classification**	**SUMOylated protein candidates**
Reported as SUMOylated	Rpl37a, Lmna
Not reported as SUMOylated	Rps9, Rpl32, Eif3e, Gsn, Stx12, Bgn, Drosha, Uqcrh, Plxnb2, Rpl18a, Atac2, Ermp1, Mrpl4, Tmsb4x, Rpsa, Lgals3, Pcolce, Tuba1b, Pbrm1, Myof, Dynlrb1, Fam63b, Taz, Rps3a, Myl9, Rpl6, Narf, Arpc1b, Psmb4, Polr1d, Rpl10, Fth1, Anxa5, Plscr3, Wisp2, Cops7a

**Table 2:** Classification of the identified SUMOylated protein candidates according to the presence of SUMO consensus recognition site or SUMO-interacting motif (SIM).

**Type of the included sequences**	**SUMOylated protein candidates**
SUMO consensus recognition site	Lmna, Rps9, Drosha, Uqcrh, Plxnb2, Rpl18a, Atac2, Ermp1, Rpsa, Tuba1b, Pbrm1, Myof, Fam63b, Rps3a, Narf, Psmb4, Anxa5
SUMO-interacting motif (SIM)	Lmna, Eif3e, Gsn, Stx12, Bgn, Drosha, Plxnb2, Atac2, Ermp1, Mrpl4, Rpsa, Lgals3, Pcolce, Tuba1b, Pbrm1, Myof, Dynlrb1, Fam63b, Rpl6, Narf, Arpc1b, Rpl10, Anxa5, Cops7a

### Confirmation of Atac2 as a novel SUMOylated protein

**Figure 5:**
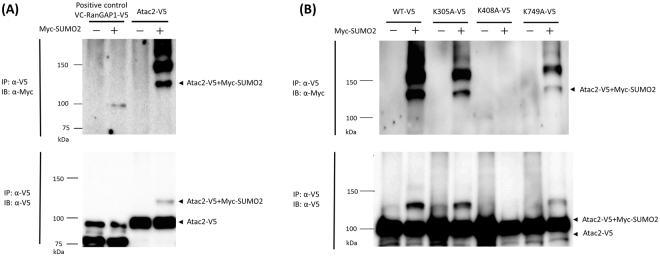
Identification of novel SUMOylated protein Atac2 and its SUMOylation site. (**A**) Atac2 is modified by SUMO2. NIH3T3 cells transfected with the indicated plasmids were subjected to immunoprecipitation with anti-V5 antibodies. The immunoprecipitated proteins were blotted with the indicated antibodies. IP: immunoprecipitation. IB: immunoblotting. The arrowheads show the expected sizes of the indicated proteins. (**B**) SUMO2 binds to K408 in Atac2. NIH3T3 cells expressing the indicated proteins were subjected to immunoprecipitation followed by Western blotting analysis.

To ensure that the candidate proteins were SUMOylated by SUMO2, the proteins were immunoprecipitated, denatured, and then analyzed by Western blotting. Because the SUMOylation by SUMO2 occurred via covalent bond conjugation, it could be distinguished from non-covalent SUMO2 interactions by the presence of upshift in size. The candidate proteins and SUMO2 were genetically tagged with V5 and Myc epitope tags, respectively, and the plasmids were introduced into NIH3T3 cells. Lysates of the cells expressing the V5-tagged proteins in the presence or absence of the Myc-SUMO2 were immunoprecipitated with anti-V5 antibodies and subjected to Western blotting analysis. Of the candidate proteins, Atac2-V5 (92 kDa) showed a clear upshifted signal (Fig. 5A) at approximately 120 kDa in an immunoblot with an anti-V5 antibody when co-expressed with Myc-SUMO2. The Myc-SUMO2 proteins were detected at the same gel location (120 kDa) by immunoblotting with an anti-Myc antibody, suggesting the co-existence of Atac2-V5 and Myc-SUMO2. The increased size (approx. 30 kDa) was slightly larger than the calculated size of Myc-SUMO2 (12 kDa). In previous reports^48–50^, the modification of a target protein with SUMOs decreased the protein’s mobility in the SDS-PAGE gel. Therefore, the observed upshifted band can be reasonably assigned to the SUMOylated Atac2. In addition to the band at approximately 120 kDa, Myc-SUMO2 proteins over 150 kDa were detected in a ladder-like arrangement; however, the co-existence of Atac2-V5 proteins was not detected by immunoblotting with the anti-V5 antibody. It is not clear whether the Myc-SUMO2 proteins that were over 150 kDa originated from Atac2 that was altered by either multiple SUMO proteins or other post-translational protein modifiers. The upshifted bands could have originated from SUMOylated proteins that co-precipitated with SUMOylated Atac2. We analyze the origin of the upshifted bands in a later section.

Next, the SUMOsp algorithm^51^, which searches for SUMO recognition sites based on the consensus sequence Ψ-K-X-E/D, was used to predict SUMOylations site in Atac2. The algorithm identified three lysine residues, K305, K408, and K749, as possible SUMOylation sites in Atac2. Cells that expressed wild-type Atac2 or Atac2 mutants (K305A, K408A, or K749A) were subjected to the same immunoprecipitation analysis as before (Fig. 5B). Bands with SUMOylated proteins were detected for wild-type Atac2 and the K305A and K749A mutants. In contrast, no Myc-SUMO2 bands larger than 120 kDa were found for the K408A mutant, indicating that the SUMOylation of the K408A mutant did not occur. To exclude the possibility that the loss of positive surface charge by the K408A mutation hampered the SUMOylation of Atac2, a K408R mutant whose net surface charge was the same as that of the wild-type protein was also generated and examined. No SUMOylation was observed for the K408R mutant (Supplementary Fig. 2). From these results, we concluded that K408 of Atac2 is a SUMOylation site that is targeted by SUMO2.

**Figure 6:**
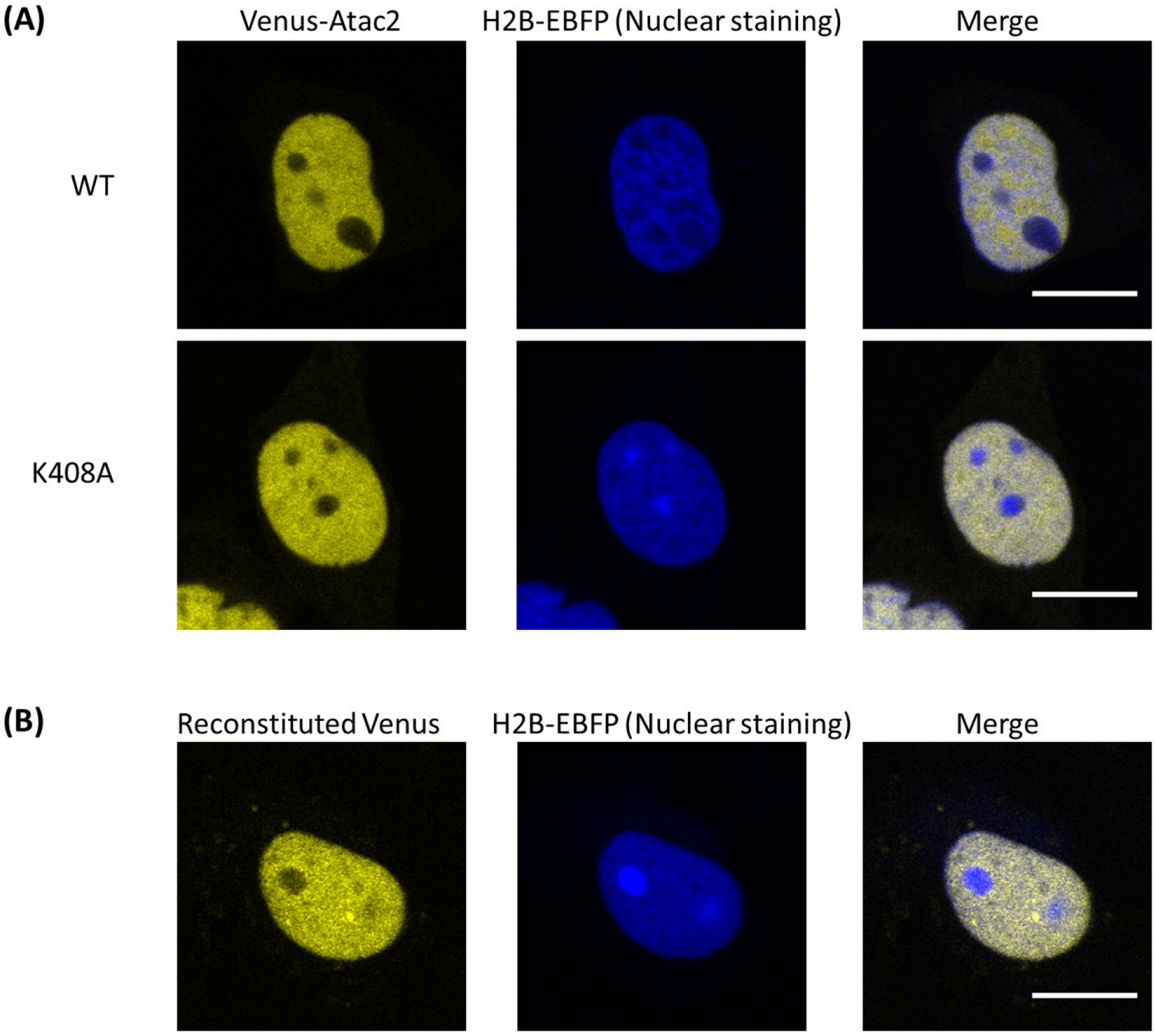
Subcellular localization of the SUMOylated Atac2. Fluorescence images were obtained from NIH3T3 cells expressing either Venus-fused wild-type or mutant K408A Atac2 co-expressed with H2B-EBFP (**A**) and from VN-SUMO2 stable cell lines co-expressing VC-Atac2 and H2B-EBFP (**B**). Scale bar: 10 μm.

Next, NIH3T3 cells that expressed Venus-fused wild-type or K408A Atac2 were observed under a confocal fluorescence microscope to determine the localization of SUMOylated Atac2 (Fig. 6A). Venus-Atac2 showed preferential localization in the nucleus, which was consistent with the previous report^52^. The Venus-K408A Atac2 mutant localized identically, indicating that SUMOylation of Atac2 does not alter its localization. Subsequently, the location of SUMOylated Atac2 was visualized using the split Venus reconstitution method. In the cells that expressed both VN-SUMO2 and VC-Atac2, fluorescence of the reconstituted Venus protein was observed in the nucleus (Fig. 6B), indicating that the SUMOylation did not perturb the Atac2 localization. In summary, Atac2 was SUMOylated by SUMO2, and the SUMOylation of Atac2 did not alter its localization.

### Modification of Atac2 by a single SUMO2 *in vitro*

**Figure 7:**
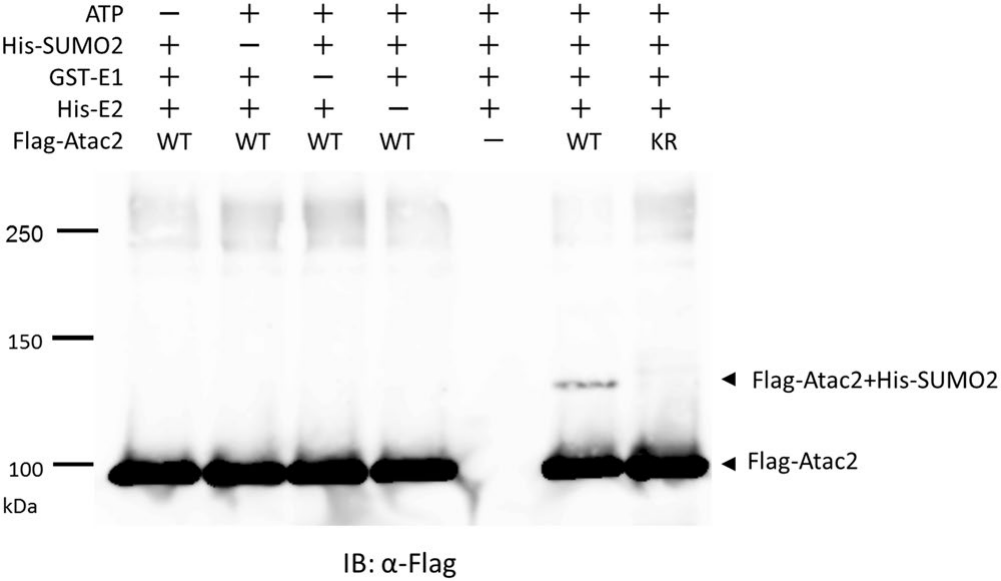
Single SUMO2 modification of Atac2 *in vitro*. Recombinant FLAG-fused Atac2 proteins, including the wild-type (WT) and the K408R (KR), were incubated in the presence or absence of each of the following components, as indicated: ATP, GST-tag purified E1, His-tag purified E2, and His-tag purified SUMO2. Reaction products were analyzed by Western blot with anti-FLAG antibodies.

SUMOylation of Atac2 was further analyzed *in vitro*. The wild-type or K408R Atac2, each of which was fused with an N-terminal FLAG epitope tag, was purified from mammalian HEK293T cells. Recombinant N-terminally GST-tagged E1 (*Mus musculus* Aos1/Uba2), His-tagged E2 (*Xenopus* Ubc9), and His-tagged human SUMO2 were purified from *E. coli*. E3 was not used because previous reports stated that *in vitro* SUMOylation proceeds without E3^53,54^. The purified FLAG-tagged Atac2 proteins (wild type and K408R) were mixed independently in the presence or absence of each of the following materials: ATP, E1, E2, and SUMO2. The mixed solutions were subjected to Western blotting analysis (Fig. 7). The upshifted SUMOylation band was detected only in the mixture that contained all the materials (ATP, E1, E2, and SUMO2) that are required for SUMOylation. On the other hand, no shifted bands were observed when FLAG-K408R Atac2 proteins were used in place of wild-type Atac2. These results demonstrated that Atac2 was monoSUMOylated *in vitro* at a lysine408.

### Modification of Atac2 by multiple SUMO2 or SUMO1 proteins

**Figure 8:**
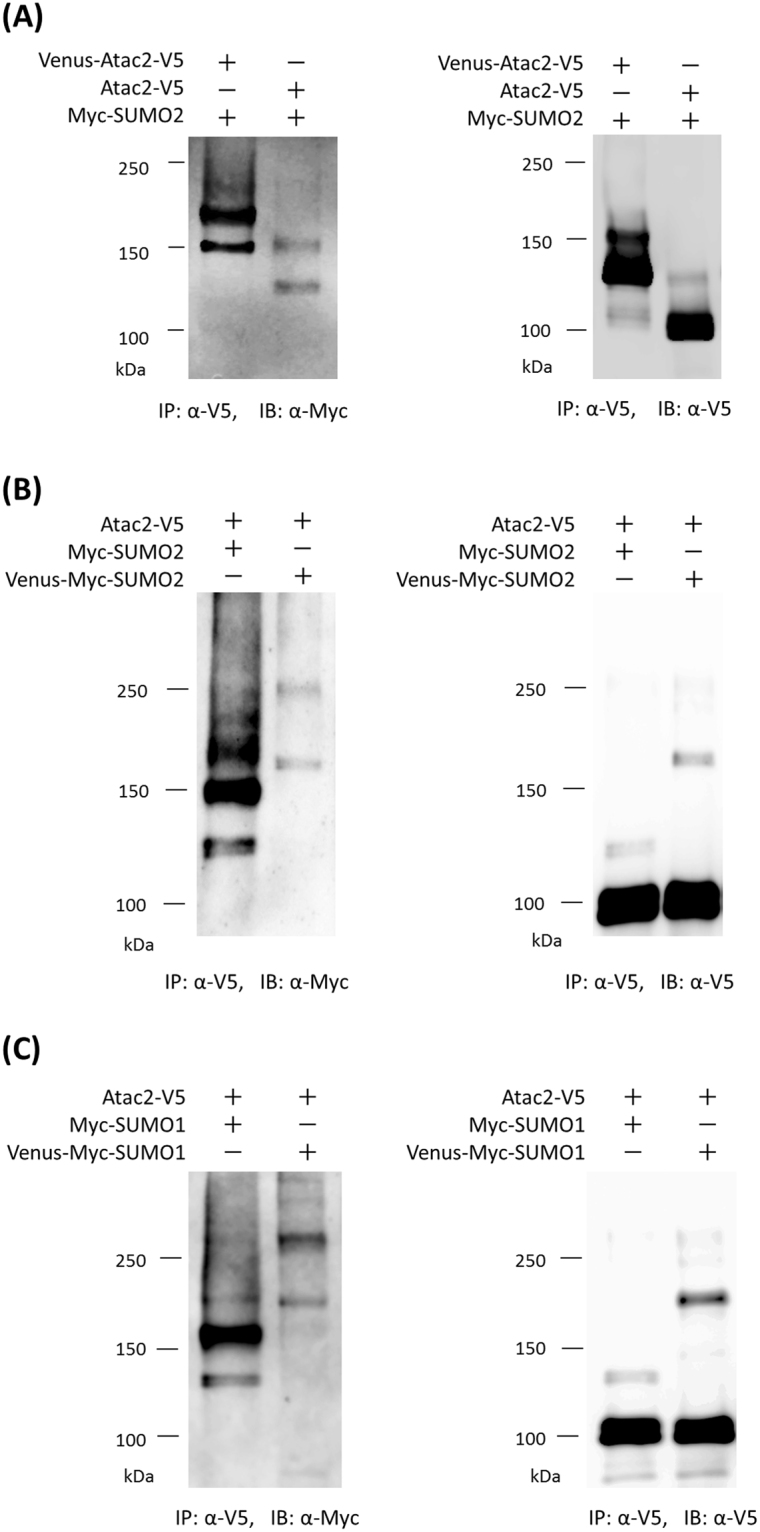
Further analyses of Atac2 SUMOylation. (**A**) Change in Atac2 size to examine the origin of the unknown Myc-SUMO2 proteins that were detected at approximately 150 kDa. NIH3T3 cells transfected with the indicated plasmids were immunoprecipitated with the indicated antibodies. The obtained samples were immunoblotted with the indicated antibodies. (**B**) Evaluation of further modification of monoSUMOylated Atac2 by Myc-SUMO2 molecules by changing SUMO2 size. NIH3T3 cells were transfected with Atac2-V5 in the presence of Myc-tagged SUMO2 or Venus-fused Myc-SUMO2 and subjected to immunoprecipitation with anti-V5 antibodies. The immunoprecipitated proteins were resolved by SDS-PAGE and analyzed by Western blotting with the indicated antibodies. (**C**) Analysis of SUMO1 modification of Atac2. NIH3T3 cells were transfected with Atac2-V5 in the presence of Myc-tagged SUMO1 or Venus-fused Myc-SUMO1 and subjected to immunoprecipitation with anti-V5 antibodies. The immunoprecipitated proteins were subjected to Western blotting with the indicated antibodies.

IP followed by Western blotting analysis was performed on the Venus-fused Atac2 (119 kDa) to compare its molecular weight with that of the Atac2 protein lacking the Venus fusion; in this manner, we hoped to clarify the origin of the strongly detected Myc-SUMO2 band that appeared at approximately 150 kDa in living cells (discussed above). In the case of Venus-Atac2 expression, the monoSUMOylated band (120 kDa) that was detected when the Atac2 protein was not fused with Venus was upshifted to 150 kDa, which was expected when the size of Venus (27 kDa) is considered (Fig. 8A). Similarly, the unknown 150 kDa Myc-SUMO2 band was upshifted to 180 kDa. Additionally, the upshifts were also observed in the experiments using Atac2 fused with the C-terminal Venus fragment, where the upshift corresponded to the size of the fused fragment (10 kDa) (Supplementary Fig. 3). From the further experiment using double-V5-tagged Atac2, where the existence of Atac2 around 150 kDa was detected with anti-V5 antibodies (Supplementary Fig. 4), the reason why single-V5-tagged Atac2 could not be blotted with anti-V5 antibodies around 150 kDa would be due to the quality of the antibodies. Taken together, these results indicated that the Myc-SUMO2 band at 150 kDa originated from SUMOylated Atac2.

Next, IP and Western blotting analysis were performed with Venus-tagged SUMO2 (Venus-Myc-SUMO2) (Fig. 8B) to determine whether the higher molecular weight band (150 kDa) was a form of Atac2 that had been SUMOylated more than once. The result revealed that the higher band of approximately 150 kDa was upshifted to approximately 250 kDa in the Venus-Myc-SUMO2 expressing cells; this upshift (100 kDa) was greater than that expected for the putative monoSUMOylation (50 kDa). These results indicated that Atac2 could be modified by two or more SUMO2 proteins.

The selectivity of SUMO proteins in the Atac2 SUMOylation process was also investigated. NIH3T3 cells that contained V5-tagged Atac2 and either Myc-SUMO1 or Venus-Myc-SUMO1 proteins were subjected to IP and Western blotting analysis (Fig. 8C). The Myc-SUMO1-modified Atac2 was detected at 120 kDa and almost 150 kDa. When Venus-tagged Myc-SUMO1 was used, SUMOylated Atac2 was detected at 150 kDa and almost 250 kDa. The detected SUMOylation patterns were the same as those of the experiments with Myc-SUMO2 and Venus-Myc-SUMO2 (shown in Fig. 8B), suggesting that Atac2 was also SUMOylated by several SUMO1 molecules. Based on these data, we concluded that Atac2 was modified by multiple SUMO proteins without a preference for a SUMO subtype.

## Discussion

In this work, we developed a method to screen for mammalian SUMOylated proteins that is based on reconstituting split fluorescent protein fragments. The method identified 36 unreported SUMOylation candidates with diverse functions and localizations. Of the candidate proteins, we confirmed that Atac2 was SUMOylated by SUMO2 at K408. Furthermore, Western blotting analysis revealed that Atac2 was modified by more than two SUMOs without specificity for a particular SUMO paralog.

The present method was based on irreversible fluorescent protein reconstitution, which would be beneficial in a SUMOylation screening. Conventional IP-MS-based screening methods have been implicated in comprehensive screening of SUMOylated proteins^20,21^ and further identifying of SUMOylation sites in various contexts^55–58^, which yielded profound insights into the biological role of SUMOylation. The IP-MS-based methods relied on the analysis of the enzymatically-digested peptides, requiring cell lysis before sample preparation. Because the deSUMOylation by SUMO proteases occurred not only in living cells but potentially after cell lysis, possibility of detecting SUMOylation could be reduced in IP-MS-based screening approaches. In contrast, the fluorescent signal generated by split fluorescent protein reconstitution maintains upon deSUMOylation, because of irreversible reaction of the reconstitution. Therefore, the present method has a potential in detecting rarely SUMOylated proteins. In addition, the present methods based on single cell analysis enabled detection of the SUMOylation signal from proteins individually, which would not be affected by signals generated by other strongly SUMOylated proteins. Under physiological condition, SUMO proteins are conjugated to substrate proteins with different modification level upon protein species. Therefore, the detectable scope of SUMOylated proteins was potentially biased by those factors in conventional IP-MS-based approaches because frequently-SUMOylated proteins are preferentially immunoprecipitated. In the present method, on the other hand, both library proteins and SUMO were conjugated with split fluorescent protein fragments and exogenously expressed, whose signals were respectively detected by FACS sorting. Therefore, SUMOylation-rate bias could be mitigated in the present SUMOylation screening method. It would be greatly helpful in detecting SUMOylation of proteins at infrequent SUMOylation rate. Of the 38 proteins detected by the present method, 24 proteins, including Atac2, were detected by the previous MS analysis^59^, supporting the reliability of the present methods as SUMOylation screening. In contrast, the rest 14 proteins have been not yet identified by the MS-based screening, indicating that the scope of detectable targets by the present split fluorescent protein reconstitution approach was partially different from that by previous MS-based approaches. Though detectable targets were limited at the present stage, it could be improved by enlarging the cDNA libraries used for the analysis. Collectively, the present system based on fluorescence protein reconstitution has unique features in the detection of SUMOylation, which could be complementally exploited for exploring wide ranges of SUMOylated proteins.

Another advantage is that the scope of candidate proteins that can be examined is not restricted by the proteins’ localization. The previous screening method, which was based on the yeast two-hybrid method^22^, required that the target proteins be recruited into the nucleus, where transcription of the reporter gene was triggered upon modification by SUMOs. Therefore, SUMOylation in other intracellular compartments, such as ER or Golgi apparatus, cannot be examined with this method. In contrast, the developed screening method can assess the SUMOylation of any intracellular proteins because the reconstitution of split fluorescent protein fragments can occur anywhere in the target cell. In the present study, DNA sequence analysis revealed that the method detected Anxa5 in the cytoplasm and nucleus^37^, Drosha in the nucleus^38^, Plscr3 in mitochondria^39^, and Tuba1b in microtubules^40^. These results demonstrate that proteins localized in diverse cellular compartments can be targeted for SUMOylation assessment by the present method.

Recently, a method to screen for yeast SUMOylation based on split fluorescent protein reconstitution has been reported^60^. The method using yeast cells was inadequate for inspecting mammalian SUMOylation because it did not reflect the complexities of the mammalian SUMOylation system: SUMO-isoform specificity and cell-type-dependent SUMOylation patterns. In contrast, the present method is potentially able to explore isoform-specific SUMOylation by changing SUMO isoforms. Also, it can be applied to explore SUMOylation in other cell types including human-derived cell lines with appropriate retrovirus infection to the human cells. Moreover, the present screening method would be easily applicable to exploring SUMOylation that is induced by such factors as UV irradiation or heat shock^31^. Hence, the present method is appropriate for detecting mammalian SUMOylation under different cellular conditions.

Though the present method enabled in principle to detect SUMOylation by the fluorescent protein reconstitution, some important SUMOylated proteins would not be detected due to the issues in the cDNA library preparation. Firstly, linker length between the proteins and the Venus fragments in the library plasmids would not be appropriate for some proteins because the relative location between the amino acid termini and SUMOylation sites differ in the SUMOylated proteins. In the present study, a short GS linker (Gly-Gly-Gly-Gly-Ser) was only used for insertion between the proteins and the Venus fragments. It would be plausible that some SUMOylated proteins failed to induce Venus reconstitution because distance between the Venus fragments was too far to be brought into close proximity with the present short linker length. Secondly, due to the present cDNA library preparation protocol, some library sequences were partially inserted into the plasmids, which may lose their SUMOylation sites. In the present protocol, the library cDNAs were transferred to the plasmids containing Venus fragment by restriction enzyme digestion. Therefore, partial DNA sequences harboring the restriction sites were inserted into the plasmids. The fact that E1/E2/E3 enzymes were not detected in the screening might be due to such issues raised above. To solve these issues, further improvements will be needed for the cDNA library preparation protocol, preparation of GS-linkers with various length, and cDNAs encoding full-length proteins fused with Venus fragments.

In addition, sorting accuracy is crucial for identifying the SUMOylated proteins. In this study, we used a conventional FACS machine, which required enrichment process to decrease false-positive rates. As a result, we obtained sibling clones limiting a variety of candidate fluorescent cells. However, the latest FACS machine with superior sorting accuracy will overcome this issue, which would decrease the number of the repetition of cell incubation and cell sorting. Also, total time required for the sorting process will be significantly improved if a new cell sorter is available.

We identified Atac2 as a novel SUMOylated protein that was modified at a lysine 408, by SUMO2. Western blotting analysis also revealed that Atac2 underwent further SUMOylation by multiple SUMO1 or SUMO2 proteins. Other SUMOylation sites might be present in Atac2 because SUMO1 does not form poly-SUMO chains^61^. The fact that K408A or K408R mutations dramatically reduced Atac2 SUMOylation suggests that K408 is a key residue in this process. The SUMOylation at K408 that we identified suggests that it may have an effect on Atac2 function. Atac2 is a component of a histone acetyltransferase complex, “ATAC”, which plays an essential role in mammalian development^62,63^. Atac2 itself has weak histone acetyltransferase activity and is indispensable for the integrity of the ATAC complex^63^. Fluorescence microscope observation revealed that SUMOylated Atac2 localized in the nucleus, suggesting a relation of SUMOylation to intranuclear activities of Atac2. Further analysis is required to clarify the influence of SUMOylation on the functions of Atac2, including gene expression that is regulated by histone acetyltransferase activity and interaction with other components in the ATAC complex. The elucidation of the consequences of Atac2 SUMOylation will provide a new clue to help understand SUMOylation-regulated nuclear events.

In conclusion, we developed a method to screen mammalian SUMOylated proteins based on reconstituting split fluorescent protein fragments and FACS sorting. The present method successfully detected SUMOylated protein candidates with distinct intracellular locations and functions in living mammalian cells. Of the candidates, we found that Atac2, a histone acetyltransferase, was SUMOylated by SUMO2 at a lysine 408. Reconstituting split fluorescent protein fragments enabled the non-destructive detection of SUMOylation under live-cell conditions without any limitation to protein localization. The cells harboring SUMOylated library proteins were discriminated by the fluorescence emission from their reconstituted fluorescent proteins and separated from non-fluorescent cells by cell sorting. The utilization of mammalian cells is fitting for detecting mammalian SUMOylation because it reflects the complex mammalian SUMOylation processes. In addition, the present method can be applied to other SUMO isoforms and different mammalian cell-types with a range of stimulants. Because of such methodological advantages, the developed method could contribute to a deeper understanding of the role of SUMOylation in diverse biological contexts, which in turn gives us new insights into the significance of post-translational modifications.

## Materials and Methods

### Plasmid construction

The cDNAs of SUMO1 and SUMO2 were amplified from human genomic DNA that was extracted from HeLa cells. The RanGAP1 cDNA was amplified from a RanGAP1 cDNA clone (Kazusa DNA Res. Inst., Japan). The cDNA of Atac2 was amplified from a *Mus musculus* brain cDNA mixture (Genostaff) using nested PCR. The cDNAs of N-terminal (amino acids 1–158) or C-terminal (amino acids 159–240) fragments of Venus were amplified from full-length cDNA. Epitope tags (V5, Myc, and FLAG) were generated using overlapping primers in the PCR reaction. Additional point mutations for Atac2 (K305A, K408A, K749A, K408R) were introduced by pairs of mutagenic complementary single-stranded DNA oligomers. The deletion mutant of RanGAP1 (∆20aaRanGAP1) was generated by amplifying N-terminal (amino acids 1–514) and C-terminal (amino acids 535–587) fragments via PCR. The PCR products were subcloned into the pcDNA4/V5 His ver. B vector (Invitrogen) for general experiments, the pMX vector^64^ for retrovirus infection, or the pTriEx-3 vector (Novagen) for *in vitro* assays. Plasmids encoding His-SUMO2, GST-E1 and His-E2 were generated as previously described^65,66^. For the preparation of plasmids encoding VC-library proteins, plasmids of a cDNA library (pAP3neo cDNA Library Mouse 10T-half, 5.3 M, RIKEN BRC DNA BANK) were digested with restriction enzymes *Eco*RI and *Not*I and transferred into the *Eco*RI and *Not*I sites in the pMX vector. The cDNA of the C-terminal Venus fragment (amino acids 159–240) was cloned into the *Bam*HI and *Eco*RI sites in the vector. Considering the frame shift, the fragment was amplified with three linkers of different length, ggcggaggcgga, ggcggaggcggag, and ggcggaggcggagg, by PCR. All primers used in this study are listed in Supplementary Table 1.

### Cell cultures and transfection

NIH3T3 and HEK293T cells were cultured in DMEM supplemented with 10% fetal bovine serum (FBS), 100 unit/mL penicillin, and 100 μg/mL streptomycin at 37 °C under 5% CO_2_. After the medium was changed to DMEM supplemented with 10% fetal bovine serum (FBS) and L-glutamine, NIH3T3 cells were transfected by using Lipofectamine 2000 Reagent (Invitrogen). Transfection of HEK293T cells was performed using PEI MAX (Polysciences, Inc.). The cells were lysed and subjected to subsequent analyses 24 hours after the transfection. To generate stable cell lines, we selected transfected NIH3T3 cells with a medium containing 500 μg/mL zeocin (Invitrogen).

### Retrovirus production and infection

PlatE cells were transfected with the plasmids for retrovirus production and cultured at 37 °C under 5% CO_2_ for 8 hours. After incubation for 24 to 48 hours, the medium that included the virus that harbored the plasmid DNAs was collected and stocked at −30 °C. Eight μg/mL polybrene was added to the medium culturing NIH3T3 cells 10 minutes before being infection with the medium stock that contained retrovirus. The infected NIH3T3 cells were incubated for one more day and subjected to subsequent FACS analysis.

### FACS analysis and sorting

VN-SUMO2 stable cell lines that were infected with a retrovirus harboring the VC-library DNAs were trypsinized and suspended in PBS after they were washed a few times with PBS. Non-infected cells were also subjected to the same procedure as a control for FACS analysis. The fluorescence intensity that was emitted from 5,000 cells was analyzed by FACS (Epics Altra, BECKMAN COULTER) following the standard procedure with an excitation wavelength of 488 nm and a measurement wavelength of 525 (±15) nm. The target fluorescent cells were sorted by FACS. The region for sorting was identified by a fluorescence intensity that was higher than the autofluorescence intensity of the control cells. The sorted cells were collected in a 1.5 mL tube. After the supernatant was removed, the cells were plated in a 6-well dish and incubated.

### Identification of candidate SUMOylated proteins

The sorted cells were plated on a 10 cm dish with a low cell density. After the cells were incubated for a few weeks, we isolated separately formed single-cell clones by picking them up with a pipette. The DNA of each incubated single-cell clone was extracted by a Wizard Genomic DNA Purification Kit (Promega). The VC-library sequences of each clone’s extracted DNA were amplified by nested PCR using the following primers: 5′-CAAAGTAGACGGCATCGCAGC-3′ and 5′-TTATGTATTTTTCCATGCCTTC-3′ (primary PCR); 5′-TTTAAGCTTGCTAGCGCCATGAAGAACGGCATCAAGGCC-3′ and 5′-TTATCGTCGACCACTGTGCTGGCGGCCGC-3′ (secondary PCR). Each PCR product was subjected to the agarose gel electrophoresis. The amplified cDNAs were purified from the individual detected bands by FastGene Gel/PCR Extraction kit (GeneTics). In case that multiple bands were detected, individual cDNAs were collected from the separated bands and purified in the same manner. All the purified cDNAs were directly sequenced with the following primer: 5′-TGGTCCTGCTGGAGTTCGTG-3′. The sequencing data were analyzed for homologies to nucleotide sequences in the GenBank database using a BLAST search. The SUMOylation sites were predicted by searching for the putative SUMO consensus sequence using the SUMOsp algorithm^51^. The SIM sequences were analyzed by the SIM algorithm^67^.

### Immunoprecipitation and Western blotting

The immunoprecipitation method was performed as previously reported^68^. Anti-V5 antibody (Invitrogen) and NP-40 lysis buffer (50 mM Tris-HCl, pH 8.0, 150 mM NaCl, and 1% NP-40) supplemented with 20 mM N-ethylmaleimide (freshly dissolved) and complete protease inhibitor (Roche) were used for the immunoprecipitation. The immunoprecipitated proteins were separated by SDS-PAGE and transferred to a nitrocellulose membrane (Amersham^TM^ Hybond^TM^-ECL, GE Healthcare). The membranes were blocked with 1% skim milk (BD) in TBS-T buffer (50 mM Tris-HCl, pH 8, 150 mM NaCl, and 0.05% Tween-20). The primary antibodies that were used were mouse monoclonal anti-V5 antibody (Invitrogen), diluted 1:5000 to 1:10000; rabbit polyclonal anti-Myc antibody (MBL), diluted 1:1000; and mouse monoclonal anti-FLAG antibody (Sigma), diluted 1:1000. The primary antibodies were visualized using the appropriate secondary antibodies, anti-mouse IgG or anti-rabbit IgG, labeled with horseradish peroxidase (Invitrogen). The immunoblot bands were detected using SuperSignal West Femto Maximum Sensitivity Substrate (Thermo Scientific), with LAS-1000 Plus image analyzers (Fuji Photo Film Co. Ltd.) or LAS 4000 mini (GE Healthcare).

### Recombinant Atac2 proteins and *in vitro* SUMOylation assay

Recombinant FLAG-Atac2 and FLAG-K408R that were expressed in HEK293T cells were purified by batch absorption using Anti-FLAG M2 Agarose Affinity Gel (Sigma) according to the manufacturer’s instructions. The bound recombinant proteins were eluted from the column by competition with 100 μg/mL FLAG peptide (Sigma) in TBS buffer (10 mM Tris-HCl and 150 mM NaCl, pH 7.4). The fractions containing FLAG fusion proteins were concentrated using Vivaspin 20-50 K (GE Healthcare), and the buffer was exchanged with the SUMOylation assay buffer (50 mM Tris-HCl, pH 7.6, 6 mM MgCl_2_, and 1 mM DTT). The recombinant His-SUMO2, GST-E1, and His-E2 were purified as previously described^65,69^. In total, 2.5 μg of FLAG-Atac2 or FLAG-K408R was reacted with or without each of the following components for 2 hours at 30 °C: 2 mM ATP (Sigma), 1.0 μg of His-SUMO2, 0.75 μg of GST-E1, and 0.05 μg of His-E2. The mixtures were subsequently analyzed by Western blotting.

### Confocal fluorescence microscopic analysis

The cells were plated on glass plates and incubated at 37 °C under 5% CO_2_ for 24 hours. Transfection was performed, and the cells were further incubated for 24 hours. The medium was exchanged with the observation buffer (DMEM modified with high glucose, L-Glutamine, HEPES, and no phenol red, supplemented with 10% FBS) before imaging. The cells were observed using an IX81-FV-1000 confocal microscope (OLYMPUS Co. Ltd.) with a UPlanSApo 100×/1.40 oil objective. EBFP and Venus were excited at 405 and 515 nm, respectively. Images were analyzed using ImageJ software.

## Electronic supplementary material

Supplementary Dataset

## References

[1] Johnson ES (2004). Protein modification by SUMO. Annu. Rev. Biochem..

[2] Melchior F (2000). SUMO–nonclassical ubiquitin. Annu. Rev. Cell Dev. Biol..

[3] Saitoh H, Hinchey J (2000). Functional heterogeneity of small ubiquitin-related protein modifiers SUMO-1 versus SUMO-2/3. J. Biol. Chem..

[4] Lapenta V (1997). SMT3A, a human homologue of the S. cerevisiae SMT3 gene, maps to chromosome 21qter and defines a novel gene family. Genomics.

[5] Chen A, Mannen H, Li SS (1998). Characterization of mouse ubiquitin-like SMT3A and SMT3B cDNAs and gene/pseudogenes. Biochem. Mol. Biol. Int..

[6] Capili AD, Lima CD (2007). Taking it step by step: mechanistic insights from structural studies of ubiquitin/ubiquitin-like protein modification pathways. Curr. Opin. Struct. Biol..

[7] Vertegaal ACO (2006). Distinct and overlapping sets of SUMO-1 and SUMO-2 target proteins revealed by quantitative proteomics. Mol. Cell. Proteomics.

[8] Tatham MH (2005). Unique binding interactions among Ubc9, SUMO and RanBP2 reveal a mechanism for SUMO paralog selection. Nat. Struct. Mol. Biol..

[9] Matunis MJ, Coutavas E, Blobel G (1996). A novel ubiquitin-like modification modulates the partitioning of the Ran-GTPase-activating protein RanGAP1 between the cytosol and the nuclear pore complex. J. Cell Biol..

[10] Zhu S (2009). Protection from isopeptidase-mediated deconjugation regulates paralog-selective sumoylation of RanGAP1. Mol. Cell.

[11] Li Y (2003). Positive and negative regulation of APP amyloidogenesis by sumoylation. Proc. Natl. Acad. Sci. USA.

[12] Geiss-friedlander R, Melchior F (2007). Concepts in sumoylation: a decade on. Nat. Rev. Mol. Cell Biol..

[13] Hickey CM, Wilson NR, Hochstrasser M (2012). Function and regulation of SUMO proteases. Nat. Rev. Mol. Cell Biol..

[14] Ouyang J, Gill G (2009). SUMO engages multiple corepressors to regulate chromatin structure and transcription. Epigenetics.

[15] Bergink S, Jentsch S (2009). Principles of ubiquitin and SUMO modifications in DNA repair. Nature.

[16] Pelisch F (2014). Dynamic SUMO modification regulates mitotic chromosome assembly and cell cycle progression in Caenorhabditis elegans. Nat. Commun..

[17] Lee J (2008). Dual modification of BMAL1 by SUMO2/3 and ubiquitin promotes circadian activation of the CLOCK/BMAL1 complex. Mol. Cell. Biol..

[18] Girdwood D (2003). P300 transcriptional repression is mediated by SUMO modification. Mol. Cell.

[19] Myatt SS (2014). SUMOylation inhibits FOXM1 activity and delays mitotic transition. Oncogene.

[20] Zhao Y, Kwon SW, Anselmo A, Kaur K, White MA (2004). Broad spectrum identification of cellular small ubiquitin-related modifier (SUMO) substrate proteins. J. Biol. Chem..

[21] Tirard M (2012). In vivo localization and identification of SUMOylated proteins in the brain of His6-HA-SUMO1 knock-in mice. Proc. Natl. Acad. Sci. USA.

[22] Hannich JT (2005). Defining the SUMO-modified proteome by multiple approaches in Saccharomyces cerevisiae. J. Biol. Chem..

[23] Takahashi Y, Toh-E A, Kikuchi Y (2003). Comparative analysis of yeast PIAS-type SUMO ligases in vivo and *in vitro*. J. Biochem..

[24] Bylebyl GR, Belichenko I, Johnson ES (2003). The SUMO Isopeptidase Ulp2 Prevents Accumulation of SUMO Chains in Yeast. J. Biol. Chem..

[25] Ji Z (2007). Regulation of the Ets-1 transcription factor by sumoylation and ubiquitinylation. Oncogene.

[26] Degerny C (2005). SUMO modification of the Ets-related transcription factor ERM inhibits its transcriptional activity. J. Biol. Chem..

[27] Isogai M (2011). Structure and characteristics of reassembled fluorescent protein, a new insight into the reassembly mechanisms. Bioorganic Med. Chem. Lett..

[28] Kerppola TK (2006). Design and implementation of bimolecular fluorescence complementation (BiFC) assays for the visualization of protein interactions in living cells. Nat. Protoc..

[29] Hu C-D, Chinenov Y, Kerppola TK (2002). Visualization of interactions among bZIP and Rel family proteins in living cells using bimolecular fluorescence complementation. Mol. Cell.

[30] Ozawa T, Sako Y, Sato M, Kitamura T, Umezawa Y (2003). A genetic approach to identifying mitochondrial proteins. Nat. Biotechnol..

[31] Tempé D, Piechaczyk M, Bossis G (2008). SUMO under stress. Biochem. Soc. Trans..

[32] Nagai T (2002). A variant of yellow fluorescent protein with fast and efficient maturation for cell-biological applications. Nat. Biotechnol..

[33] Mahajan R, Delphin C, Guan T, Gerace L, Melchior F (1997). A small ubiquitin-related polypeptide involved in targeting RanGAP1 to nuclear pore complex protein RanBP2. Cell.

[34] Macauley MS (2004). Structural and dynamic independence of isopeptide-linked RanGAP1 and SUMO-1. J. Biol. Chem..

[35] Matunis MJ, Wu J, Blobel G (1998). SUMO-1 modification and its role in targeting the Ran GTPase-activating protein, RanGAP1, to the nuclear pore complex. J. Cell Biol..

[36] Rodriguez MS, Dargemont C, Hay RT (2001). SUMO-1 conjugation *in vivo* requires both a consensus modification motif and nuclear targeting. J. Biol. Chem..

[37] Sun J, Salem HH, Bird P (1992). Nucleolar and cytoplasmic localization of annexin V. FEBS Lett..

[38] Tang X, Zhang Y, Tucker L, Ramratnam B (2010). Phosphorylation of the RNase III enzyme Drosha at Serine300 or Serine302 is required for its nuclear localization. Nucleic Acids Res..

[39] Liu J, Chen J, Dai Q, Lee RM (2003). Phospholipid scramblase 3 is the mitochondrial target of protein kinase C delta-induced apoptosis. Cancer Res..

[40] Azakir BA, Fulvio SDi, Therrien C, Sinnreich M (2010). Dysferlin interacts with tubulin and microtubules in mouse skeletal muscle. PLoS One.

[41] Yamada M (2006). NARF, an nemo-like kinase (NLK)-associated ring finger protein regulates the ubiquitylation and degradation of T cell factor/lymphoid enhancer factor (TCF/LEF). J. Biol. Chem..

[42] Bernatchez PN (2007). Myoferlin regulates vascular endothelial growth factor receptor-2 stability and function. J. Biol. Chem..

[43] Molli PR (2010). Arpc1b, a centrosomal protein, is both an activator and substrate of Aurora A. J. Cell Biol..

[44] Lei Q (2008). TAZ Promotes Cell Proliferation and Epithelial-Mesenchymal Transition and Is Inhibited by the Hippo Pathway. Mol. Cell. Biol..

[45] Yun C (2008). Nucleolar protein B23/nucleophosmin regulates the vertebrate SUMO pathway through SENP3 and SENP5 proteases. J. Cell Biol..

[46] Zhang Y-Q, Sarge KD (2008). Sumoylation regulates lamin A function and is lost in lamin A mutants associated with familial cardiomyopathies. J. Cell Biol..

[47] Hecker CM, Rabiller M, Haglund K, Bayer P, Dikic I (2006). Specification of SUMO1- and SUMO2-interacting motifs. J. Biol. Chem..

[48] Hietakangas V (2003). Phosphorylation of serine 303 is a prerequisite for the stress-inducible SUMO modification of heat shock factor 1. Mol. Cell. Biol..

[49] Subramanian L, Benson MD, Iñiguez-Lluhí JA (2003). A synergy control motif within the attenuator domain of CCAAT/enhancer-binding protein alpha inhibits transcriptional synergy through its PIASy-enhanced modification by SUMO-1 or SUMO-3. J. Biol. Chem..

[50] Eloranta JJ, Hurst HC (2002). Transcription factor AP-2 interacts with the SUMO-conjugating enzyme UBC9 and is sumolated in vivo. J. Biol. Chem..

[51] Xue Y, Zhou F, Fu C, Xu Y, Yao X (2006). SUMOsp: a web server for sumoylation site prediction. Nucleic Acids Res..

[52] Ma Y (2017). The CSRP2BP histone acetyltransferase drives smooth muscle gene expression. Nucleic Acids Res..

[53] Okuma T, Honda R, Ichikawa G, Tsumagari N, Yasuda H (1999). In vitro SUMO-1 modification requires two enzymatic steps, E1 and E2. Biochem. Biophys. Res. Commun..

[54] Desterro JM, Rodriguez MS, Kemp GD, Hay RT (1999). Identification of the enzyme required for activation of the small ubiquitin-like protein SUMO-1. J. Biol. Chem..

[55] Lamoliatte F (2014). Large-scale analysis of lysine SUMOylation by SUMO remnant immunoaffinity profiling. Nat. Commun..

[56] Hendriks IA (2017). Site-specific mapping of the human SUMO proteome reveals co-modification with phosphorylation. Nat. Struct. Mol. Biol..

[57] Lamoliatte F, McManus FP, Maarifi G, Chelbi-Alix MK, Thibault P (2017). Uncovering the SUMOylation and ubiquitylation crosstalk in human cells using sequential peptide immunopurification. Nat. Commun..

[58] Hendriks IA (2014). Uncovering global SUMOylation signaling networks in a site-specific manner. Nat. Struct. Mol. Biol..

[59] Hendriks IA, Vertegaal ACO (2016). A comprehensive compilation of SUMO proteomics. Nat. Rev. Mol. Cell Biol..

[60] Sung M-K (2013). Genome-wide bimolecular fluorescence complementation analysis of SUMO interactome in yeast. Genome Res..

[61] Gareau JR, Lima CD (2010). The SUMO pathway: emerging mechanisms that shape specificity, conjugation and recognition. Nat. Rev. Mol. Cell Biol..

[62] Wang Y-L, Faiola F, Xu M, Pan S, Martinez E (2008). Human ATAC Is a GCN5/PCAF-containing acetylase complex with a novel NC2-like histone fold module that interacts with the TATA-binding protein. J. Biol. Chem..

[63] Guelman S (2009). The Double-Histone-Acetyltransferase Complex ATAC Is Essential for Mammalian Development. Mol. Cell. Biol..

[64] Onishi M (1996). Applications of retrovirus-mediated expression cloning. Exp. Hematol..

[65] Uchimura Y, Nakamura M, Sugasawa K, Nakao M, Saitoh H (2004). Overproduction of eukaryotic SUMO-1- and SUMO-2-conjugated proteins in Escherichia coli. Anal. Biochem..

[66] Uchimura Y, Nakao M, Saitoh H (2004). Generation of SUMO-1 modified proteins in E. coli: towards understanding the biochemistry/structural biology of the SUMO-1 pathway. FEBS Lett..

[67] Zhao Q (2014). GPS-SUMO: a tool for the prediction of sumoylation sites and SUMO-interaction motifs. Nucleic Acids Res..

[68] Sarge KD, Park-Sarge O-K (2009). Detection of proteins sumoylated in vivo and *in vitro*. Methods Mol. Biol..

[69] Fukuda I (2009). Ginkgolic Acid Inhibits Protein SUMOylation by Blocking Formation of the E1-SUMO Intermediate. Chem. Biol..

