# Supplementary material for: A genetic screen to discover SUMOylated proteins in living mammalian cells
Source: Sci Rep. 2017 Dec 12;7:17443. doi: 10.1038/s41598-017-17450-7 (PMC5727073; doi:10.1038/s41598-017-17450-7)
Supplement: Supplementary file 1 — Supplementary Dataset [file 41598_2017_17450_MOESM1_ESM.doc]

**Supplementary Information**

**A genetic screening to discover SUMOylated proteins in living mammalian cells**

Maki Komiya^a^, A. Ito^b, c^, M. Endo^a^, D. Hiruma^a^, M. Hattori^a, †^, H. Saitoh^d^, M. Yoshida^b, c^, T. Ozawa^a, *^

a. Department of Chemistry, Graduate School of Science, The University of Tokyo, 7-3-1 Hongo, Bunkyo-ku, Tokyo 113-0033, Japan.

b. Chemical Genetics Laboratory, RIKEN, 2-1 Hirosawa, Wako, Saitama 351-0198, Japan

c. Chemical Genomics Research Group, RIKEN Center for Sustainable Resource Science, 2-1 Hirosawa, Wako, Saitama 351-0198, Japan

d. Department of Biological Sciences, Graduate School of Science and Technology, Kumamoto University, 2-39-1 Kurokami, Kumamoto 860-8555, Japan.

*. Correspondence should be addressed: T.O.

†. Present address: Department of Biomolecular Science and Engineering, The Institute of Scientific & Industrial Research, Osaka University, 8-1 Mihogaoka, Ibaraki, Osaka, 567-0047, Japan

Email: [ozawa@chem.s.u-tokyo.ac.jp](mailto:ozawa@chem.s.u-tokyo.ac.jp)

Tel.: 81-3-5841-4351

Fax: 81-3-5802-2989


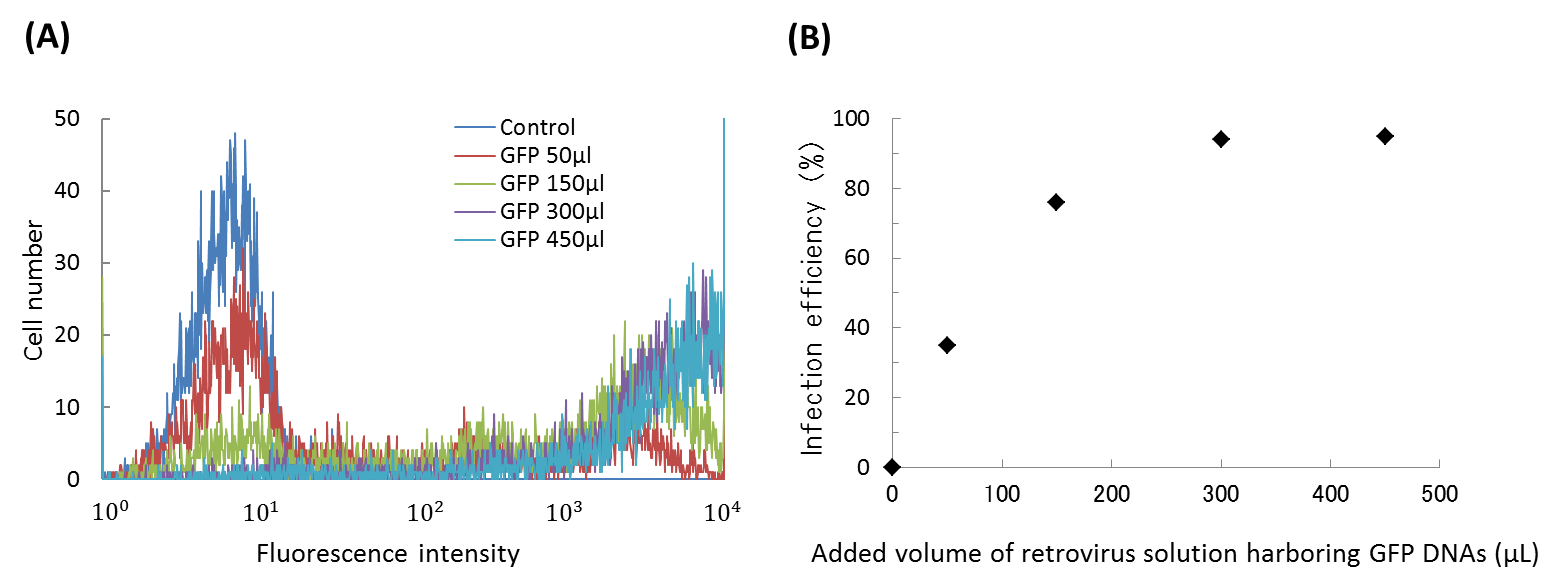


**Supplementary Fig.1. Control infection with retroviruses harboring GFP DNAs.**

(A) Fluorescence intensities of the VN-SUMO2 stable cell lines with or without retrovirus infection. The volume of added GFP-harboring retrovirus solutions was indicated. (B) The infection efficiency calculated from Supplementary Fig. 1A was estimated by the ratio of the cells with higher fluorescent intensities than those of the non-infected cells to whole cells. The number of the whole cells was 5,000.


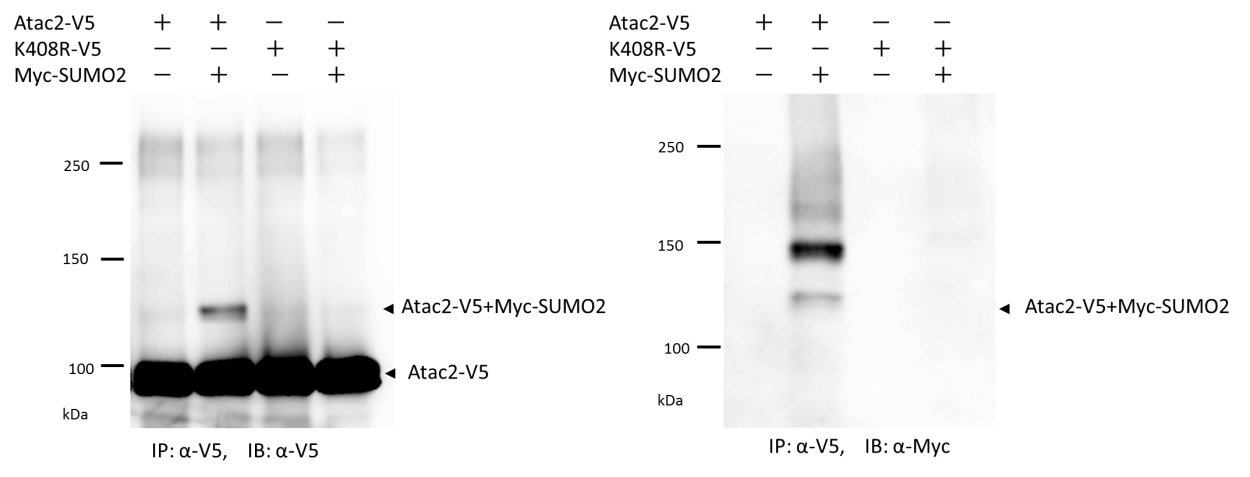


**Supplementary Fig.2. K408R mutant of Atac2 is not SUMOylated.**

NIH3T3 cells were transfected with V5-tagged wild type Atac2 or the K408R mutant in the absence or presence of Myc-SUMO2 expression. Proteins were immunoprecipitated with anti-V5 antibodies, and analyzed by Western blot with anti-V5 antibodies (Left figure) and anti-Myc antibodies (Right figure).


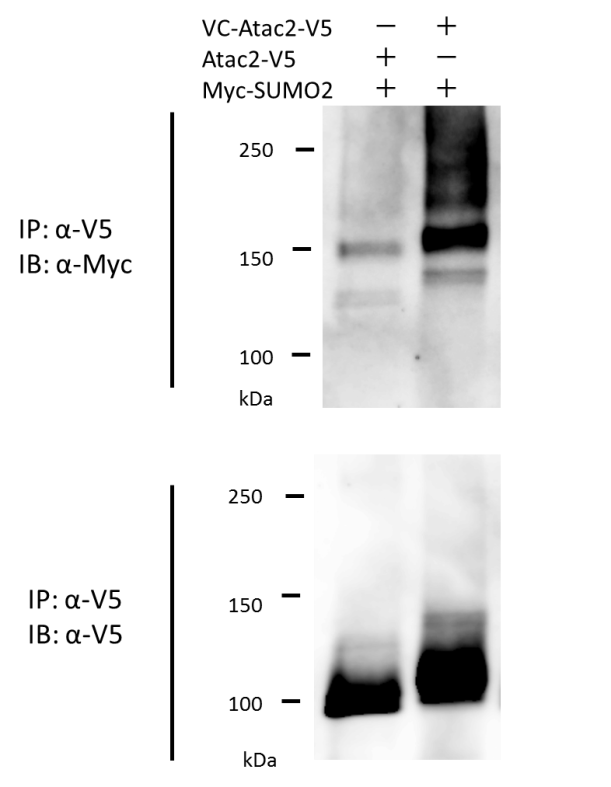


**Supplementary Fig.3.** **Examination of the origin of the unknown Myc-SUMO2 proteins detected around 150 kDa by using VC- fused Atac2.**

NIH3T3 cells transfected with Atac2-V5 or VC-tagged Atac2-V5 in the presence of Myc-SUMO2 expression were immunoprecipitated with anti-V5 antibodies. The obtained samples were immunoblotted with anti-Myc antibodies (Top figure) and anti-V5 antibodies (Bottom figure).


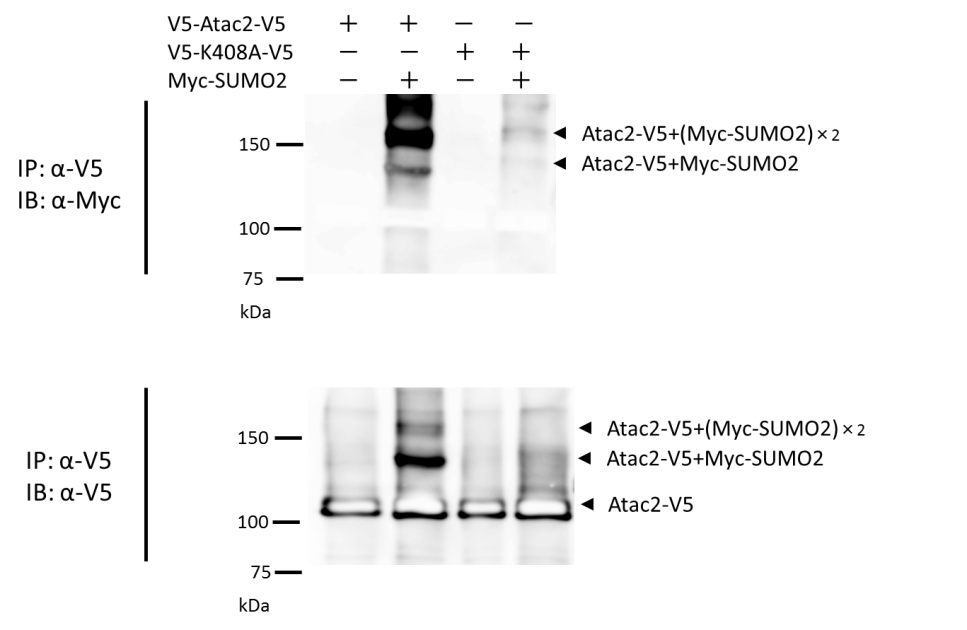


**Supplementary Fig.4. Detection of the 150 kDa band with anti-V5 antibody by using double-V5-tagged Atac2.**

NIH3T3 cells transfected with indicated plasmids were immunoprecipitated with anti-V5 antibodies. The obtained proteins were immunoblotted with indicated antibodies.


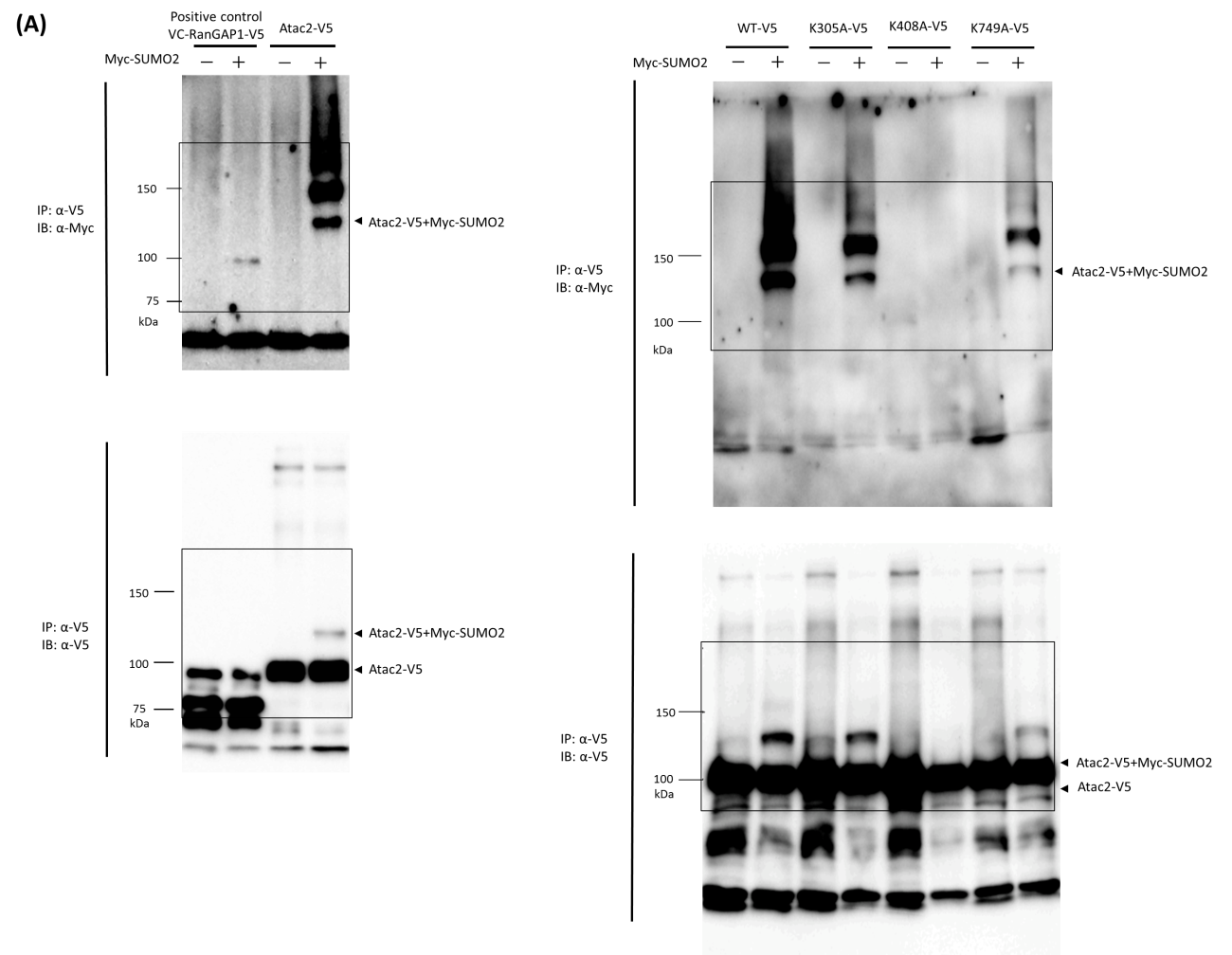


**
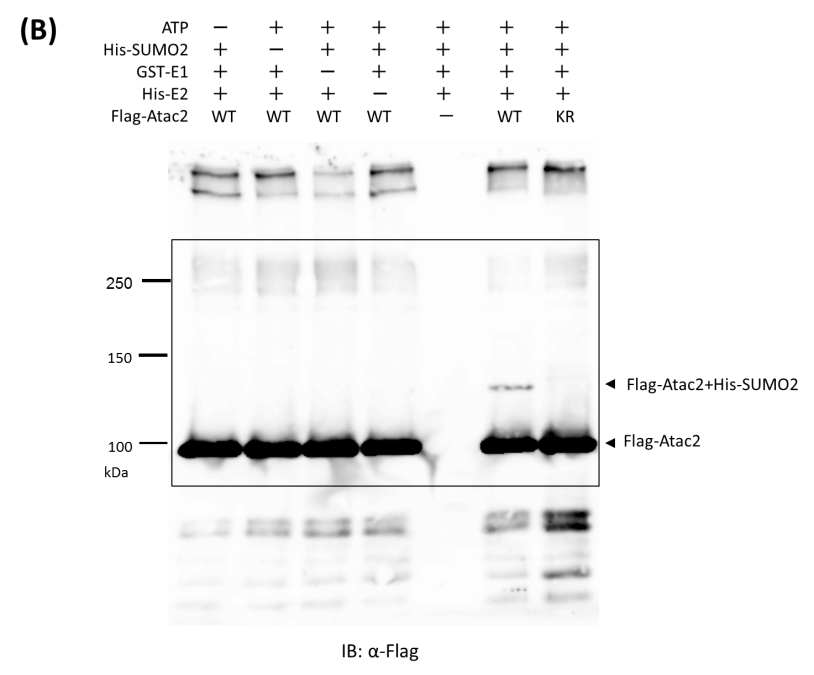
**

**
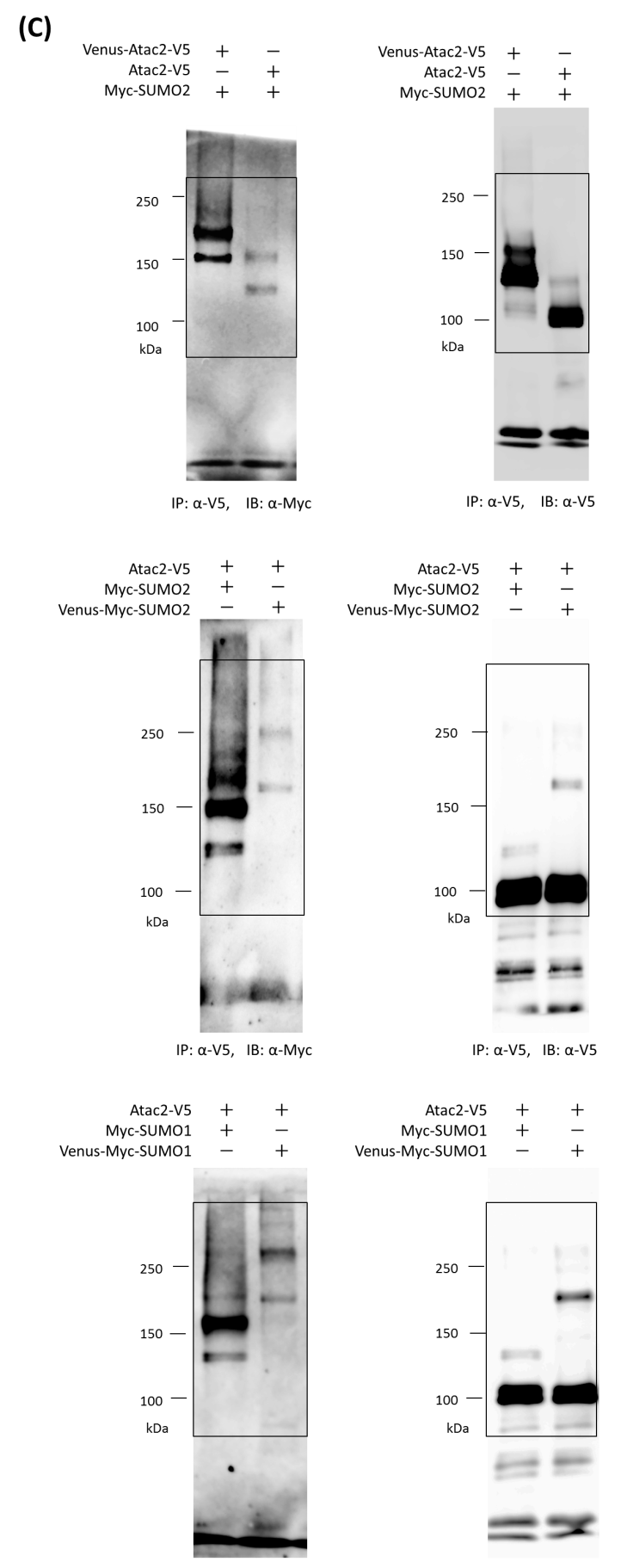
**

**
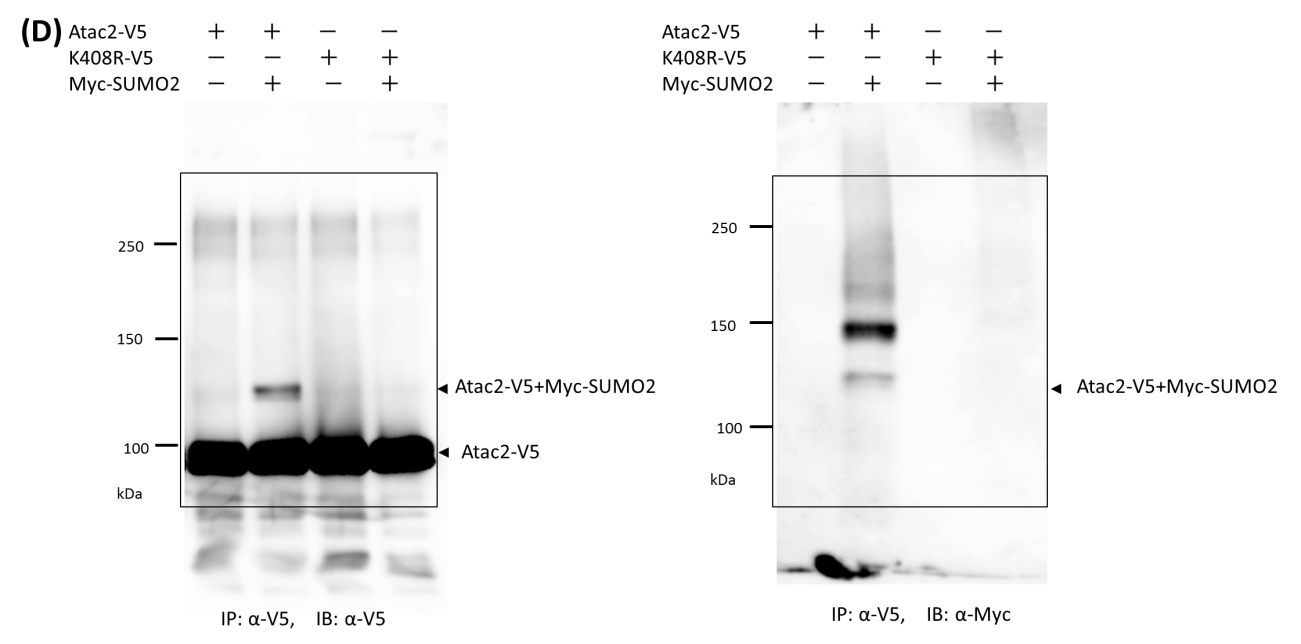
**

**
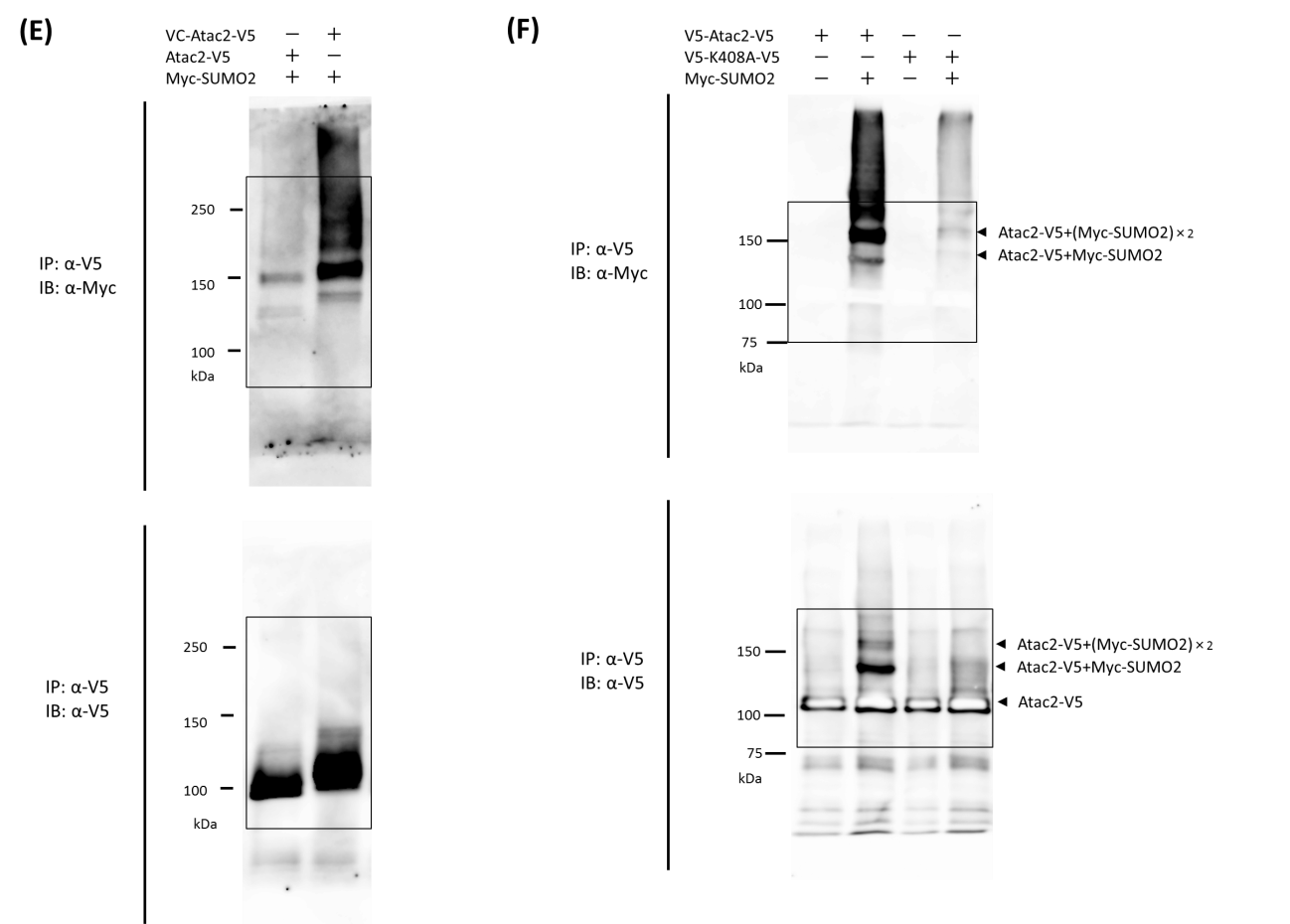
**

**Supplementary Fig.5. Full blots images.**

(A) Full blots images of **Fig. 5**. (B) Full blots images of **Fig. 7**. (C) Full blots images of **Fig. 8**. (D) Full blots images of **Supplementary Fig. 2**. (E) Full blots images of **Supplementary Fig. 3**. (F) Full blots images of **Supplementary Fig. 4**.

Supplementary Table. 1. The list of primers used in this study.

| **Primer name** | **Sequences (5'→3')** |
| --- | --- |
| VC-Forward | TTTGGATCCGCTAGCGCCATGAAGAACGGCATCAAG |
| VC-Reverse-1 | GGTGAATTCTCCGCCTCCGCCCTTGTACAGCTCGTCC |
| VC-Reverse-2 | GGTGAATTCCTCCGCCTCCGCCCTTGTACAGCTCGTCC |
| VC-Reverse-3 | GGTGAATTCCCTCCGCCTCCGCCCTTGTACAGCTCGTCC |
| VN-Forward | AGTGCGGCCGCTTAAGCTTACCATGGTGAGCAAGGGCGAG |
| VN-Reverse | CATGGATCCGGCTCCGCCCTGCTTGTCGGCGGTGATATAG |
| Venus-Forward for Venus-Atac2 construction | TTTGCTAGCATGGTGAGCAAGGGCG |
| Venus-Reverse for Venus-Atac2 construction | TTTGAATTCTCCGCCTCCGCCCTTGTACAGCTCGTCCATGCCG |
| VC-Forward for VC-Atac2 construction | TTTGCTAGCATGAAGAACGGCAT |
| VC-Reverse for VC-Atac2 construction | TTTGAATTCTCCGCCTCCGCCCTTGTAC |
| Venus-Forward for Venus-Myc-SUMO1/2 construction | TTTGCTAGCATGGTGAGCAAGGGCGAGGAGCTGTTCAC |
| Venus-Reverse for Venus-Myc-SUMO1/2 construction | TTTGCTAGCTCCGCCTCCGCCCTTGTACAGCTCGTCCA |
| SUMO1-Forward | TTTAAGCTTATGTCTGACCAGGAGGCAAAACCTTCAACT |
| SUMO1-Reverse | TTTCTCGAGCTAAACTGTTGAATGACCCCCCGTTTGTTC |
| SUMO2-Forward | GGCGGATCCATGGCCGACGAAAAGCCCAAG |
| SUMO2-Reverse | CCCTCTAGACTCGAGCGGTAGACACCTCCCGTCTGCTG |
| Myc-SUMO1-Forward | GGCGCTAGCACCATGGAACAAAAACTCATCTCAGAAGAGGATCTGAAGCTTATGTCTGACCAGGAGGCAAAACCTT |
| Myc-SUMO1-Reverse | TTTCTCGAGCTAAACTGTTGAATGACCCCCCGTTTGTTC |
| Myc-SUMO2-Forward | GGCGCTAGCACCATGGAACAAAAACTCATCTCAGAAGAGGATCTGAAGCTTATGGCCGACGAAAAGCCCAAG |
| Myc-SUMO2-Reverse | TTTCTCGAGCTAGTAGACACCTCCCGTCTGCTGTTGGAA |
| RanGAP1-Forward | TTTGGATCCGCCACCATGGCCTCGGAAGACATTGCCAAGCTGGCA |
| RanGAP1-Reverse | AAACTCGAGCTAGACCTTGTACAGCGTCTGCAGCAGACT |
| RanGAP1(1 to 514)-Forward | TTTGGATCCGCCACCATGGCCTCGGAAGACATTGCCAAGCTGGCA |
| RanGAP1(1 to 514)-Reverse | AAAGAATTCAGATCCACCTCCGCCGGTGAGGAAGGTGTTGGA |
| RanGAP1(535-587)-Forward | TTTGAATTCGGCGGAGGTGGATCTCTGTACGGCCCACTGATG |
| RanGAP1(535-587)-Reverse | AAACTCGAGCTAGACCTTGTACAGCGTCTGCAGCAGACT |
| Atac2-primary-Forward | AAGGTCTGGGGGATCCCGGCAGCTTAGGAGTGCGTGTGAGCAGG |
| Atac2-primary-Reverse | CACAGGAGACCAGGGCCCACTTCGAGGATAAGGGCCTGCAGGGC |
| Atac2-secondary-Forward | TTTGAATTCATGGATAGTAGCATCCACCTGAGTGGGCTCCTCAG |
| Atac2-secondary-Reverse | TTTCTCGAGTCTAGACGGCGTCGGAGCCTCAGGAAGAATGCATG |
| Flag-Atac2-Forward | TTTGAATTCCGACTACAAGGACGATGACGACAAGGGCGATAGTA |
| Flag-Atac2-Reverse | TTTGCGGCCGCTCAGCGTCGGAGCCTCAGGAAGAATGCATGCTT |
| Atac2 K305A-Forward | TCTGATTTTGGAGGCAGGAGAAGTGATCG |
| Atac2 K305A-Reverse | CGATCACTTCTCCTGCCTCCAAAATCAGA |
| Atac2 K408A-Forward | CCGGAACAGATAGCACAGGAAGTGGAC |
| Atac2 K408A-Reverse | GTCCACTTCCTGTGCTATCTGTTCCGG |
| Atac2 K749A-Forward | GTTTGGCTTCGCGACCGAGGAGTAT |
| Atac2 K749A-Reverse | ATACTCCTCGGTCGCGAAGCCAAAC |
| Atac2 K408R-Forward | CGGAACAGATAAGACAGGAAGTGGA |
| Atac2 K408R-Reverse | TCCACTTCCTGTCTTATCTGTTCCG |

Supplementary Table. 2. SUMO consensus recognition sites, non-SUMO consensus recognition sites, and SIMs predicted by GPS-SUMO.

| **Protein** | **SUMO consensus recognition sites predicted by GPS-SUMO※1  2.0 online service** | **SUMO non-consensus recognition sites predicted by GPS-SUMO 2.0 online service** | **SIMs  predicted by GPS-SUMO 2.0 online service** |  |
| --- | --- | --- | --- | --- |
| Rpl37a |  | V**K**SA(79-82), L**K**EL(86-89), L**K**DQ(89-92) |  |  |
| Lmna | L**K**EE(88-91), A**K**LD(157-160) | A**K**LR(206-209), R**K**LE(307-310) | VVTIW(382-386) |  |
| Rps9 | M**K**LD(92-95), L**K**IE(100-103) | N**K**RE(39-42) |  |  |
| Rpl32 |  |  |  |  |
| Eif3e |  |  | IIDLF(232-236) |  |
| Gsn |  | E**K**FD(72-75), E**K**TE(717-720) | ILDLG(215-219), ITVVR(742-746) |  |
| Stx12 |  | T**K**QD(52-55), S**K***(273, 274, stop codon) | LELIK(178-182), ILDVN(195-199), ILVLV(253-257), VVVIW(266-270) |  |
| Bgn |  | Y**K**K*(367-369, stop codon), K**K***(368, 369, stop codon) | LVLVN(120-124), LVEIP(151-155), LVELR(162-166) |  |
| Drosha | F**K**GE(1303-1306) | E**K**EA(388-391), D**K**LD(445-448), T**K**LD(470-473), R**K**YR(1345-1348), I**K**K*(1371-1373, stop codon), K**K***(1372, 1373, stop codon) | ILELY(655-659), LINIM(944-948) |  |
| Uqcrh | P**K**EE(16-19) | R**K**ML(7-10), F**K**NL(85-88), L**K***(88, 89, stop codon) |  |  |
| Plxnb2 | I**K**QD(442-445), V**K**AD(833-836), P**K**PD(1512-1515) | G**K**LD(1304-1307), N**K**VT(1837-1840) | IIVVS(1216-1220), LSVIA(1716-1720) |  |
| Rpl18a | M**K**VE(127-130) | T**K**RP(169-172) |  |  |
| Atac2 | L**K**GD(96-99), I**K**QE(407-410), F**K**TE(748-751) | E**K**GE(304-307), E**K**PD(416-419), C**K**HA(769-772) | LLIVE(37-41), IITVE(236-240), VVVLY(673-677) | **※2** |
| Ermp1 | V**K**LE(182-185), V**K**RD(697-700) |  | ILAVL(369-373), LLVIA(401-405), IIAVF(464-468), LLVCS(658-662) |  |
| Mrpl4 |  |  | LDIVH(93-97), VLLVD(216-220) |  |
| Tmsb4x |  | D**K**PD(3-6), E**K**FD(11-14), K**K**TE(19-22), E**K**QA(38-41) |  |  |
| Rpsa | M**K**EE(10-13) | E**K**EE(211-214) | IYIIN(46-50), IVAIE(65-69), VSVIS(74-78), LLVVT(121-125) |  |
| Lgals3 |  |  | LITIM(145-149), IVLDF(159-163), VIVCN(184-188) |  |
| Pcolce |  | G**K**FD(202-205), R**K**CP(458-461) | VIMLT(196-200) |  |
| Tuba1b | V**K**CD(303-306) | E**K**DY(429-432) | IIDLV(114-118) |  |
| Pbrm1 | L**K**ME(101-104), L**K**RE(934-937), F**K**SD(1033-1036), A**K**KE(1387-1390) | Q**K**GE(153-156), K**K**AE(283-286), K**K**KE(483-486), R**K**EL(637-640), E**K**EK(918-921), E**K**KE(920-923), E**K**LP(923-926), E**K**RE(939-942), E**K**SE(944-947), D**K**GD(1120-1123), A**K**FA(1329-1332), K**K**AE(1439-1442) | IILEP(576-580), IVCIE(992-996), VGVLG(1576-1580) |  |
| Myof | F**K**DE(28-31), L**K**RE(881-884), F**K**LE(1079-1082), P**K**EE(1351-1354), A**K**KE(1851-1854), P**K**LD(1981-1984) | K**K**VD(36-39), E**K**RD(332-335), T**K**ND(449-452), K**K**LE(539-542), N**K**FD(591-594), R**K**KD(1014-1017), E**K**GP(1095-1098), G**K**SD(1493-1496) | VSVIF(24-28), LVIVV(65-69), IDLVI(118-122), IISIR(260-264), LLVVE(549-553), VVTLT(627-631), LLEIE(738-742), IIIWM(762-766), IEILA(1293-1297), VIEIE(1416-1420), LEVLN(1957-1961) |  |
| Dynlrb1 |  | K**K**NE(74-77) | IIVVN(20-24), LIVIQ(88-92) |  |
| Fam63b | W**K**EE(228-231) | D**K**EK(588-591), E**K**EK(590-593), E**K**EK(592-595), E**K**NS(594-597) | VIL(599-601) |  |
| Taz |  |  |  |  |
| Rps3a | P**K**FE(221-224) | S**K**KD(26-29) |  |  |
| Myl9 |  | D**K**ED(50-53), L**K**HG(163-166), D**K**DD(169-172) |  |  |
| Rpl6 |  | T**K**VE(85-88) | LIILT(158-162), LLLVT(180-184) |  |
| Narf | MKCE(1-4) | S**K**TD(15-18), E**K**GE(42-45), K**K**LE(237-240), I**K**W*(460-462, stop codon) | VEVLA(375-379) |  |
| Arpc1b |  | V**K**SL(359-362), L**K**DL(366-369), L**K**IK(369-372), I**K***(371, 372, stop codon) | LVILR(90-94), VISIC(119-123), ISVLS(332-336) |  |
| Psmb4 | V**K**FD(59-62) |  |  |  |
| Polrld |  | R**K**AI(9-12), D**K**CD(117-120) |  |  |
| Rpl10 |  |  | VIRIN(96-100) |  |
| Fth1 |  | D**K**ND(124-127) |  |  |
| Anxa5 | L**K**SE(67-70),  I**K**GD(298-301) | G**K**FE(73-76),  L**K**WG(183-186),  Y**K**KA(306-309),  K**K**AL(307-310) | LVVLL(151-155),  IRVVV(268-272),  LLLLC(310-314) |  |
| Plscr3 |  | T**K**DE(227-230), E**K**RG(284-287) |  |  |
| Wisp2 |  |  |  |  |
| Cops7a |  | A**K**IW(268-271) | LVIEA(131-135) |  |

* indicates stop codon.

The character "**K**" with bold highlight indicates the predicted SUMOylation site.

The numerals in the table indicate the position of amino acids.

※1: GPS-SUMO is updated version of SUMOsp.

※2: In the section of "Confirmation of Atac2 as a novel SUMOylated protein" in the main

manuscript, SUMOylation sites in Atac2 were predicted by old version of SUMOsp 1.0.
